# Search for new physics in events with a leptonically decaying Z boson and a large transverse momentum imbalance in proton–proton collisions at $\sqrt{s}$ = 13$\,\text {TeV}$

**DOI:** 10.1140/epjc/s10052-018-5740-1

**Published:** 2018-04-11

**Authors:** A. M. Sirunyan, A. Tumasyan, W. Adam, F. Ambrogi, E. Asilar, T. Bergauer, J. Brandstetter, E. Brondolin, M. Dragicevic, J. Erö, A. Escalante Del Valle, M. Flechl, M. Friedl, R. Frühwirth, V. M. Ghete, J. Grossmann, J. Hrubec, M. Jeitler, A. König, N. Krammer, I. Krätschmer, D. Liko, T. Madlener, I. Mikulec, E. Pree, N. Rad, H. Rohringer, J. Schieck, R. Schöfbeck, M. Spanring, D. Spitzbart, W. Waltenberger, J. Wittmann, C.-E. Wulz, M. Zarucki, V. Chekhovsky, V. Mossolov, J. Suarez Gonzalez, E. A. De Wolf, D. Di Croce, X. Janssen, J. Lauwers, M. Van De Klundert, H. Van Haevermaet, P. Van Mechelen, N. Van Remortel, S. Abu Zeid, F. Blekman, J. D’Hondt, I. De Bruyn, J. De Clercq, K. Deroover, G. Flouris, D. Lontkovskyi, S. Lowette, I. Marchesini, S. Moortgat, L. Moreels, Q. Python, K. Skovpen, S. Tavernier, W. Van Doninck, P. Van Mulders, I. Van Parijs, D. Beghin, B. Bilin, H. Brun, B. Clerbaux, G. De Lentdecker, H. Delannoy, B. Dorney, G. Fasanella, L. Favart, R. Goldouzian, A. Grebenyuk, A. K. Kalsi, T. Lenzi, J. Luetic, T. Maerschalk, A. Marinov, T. Seva, E. Starling, C. Vander Velde, P. Vanlaer, D. Vannerom, R. Yonamine, F. Zenoni, T. Cornelis, D. Dobur, A. Fagot, M. Gul, I. Khvastunov, D. Poyraz, C. Roskas, S. Salva, M. Tytgat, W. Verbeke, N. Zaganidis, H. Bakhshiansohi, O. Bondu, S. Brochet, G. Bruno, C. Caputo, A. Caudron, P. David, S. De Visscher, C. Delaere, M. Delcourt, B. Francois, A. Giammanco, M. Komm, G. Krintiras, V. Lemaitre, A. Magitteri, A. Mertens, M. Musich, K. Piotrzkowski, L. Quertenmont, A. Saggio, M. Vidal Marono, S. Wertz, J. Zobec, W. L. Aldá Júnior, F. L. Alves, G. A. Alves, L. Brito, M. Correa Martins Junior, C. Hensel, A. Moraes, M. E. Pol, P. Rebello Teles, E. Belchior Batista Das Chagas, W. Carvalho, J. Chinellato, E. Coelho, E. M. Da Costa, G. G. Da Silveira, D. De Jesus Damiao, S. Fonseca De Souza, L. M. Huertas Guativa, H. Malbouisson, M. Melo De Almeida, C. Mora Herrera, L. Mundim, H. Nogima, L. J. Sanchez Rosas, A. Santoro, A. Sznajder, M. Thiel, E. J. Tonelli Manganote, F. Torres Da Silva De Araujo, A. Vilela Pereira, S. Ahuja, C. A. Bernardes, T. R. Fernandez Perez Tomei, E. M. Gregores, P. G. Mercadante, S. F. Novaes, Sandra S. Padula, D. Romero Abad, J. C. Ruiz Vargas, A. Aleksandrov, R. Hadjiiska, P. Iaydjiev, M. Misheva, M. Rodozov, S. Shopova, G. Sultanov, A. Dimitrov, L. Litov, B. Pavlov, P. Petkov, W. Fang, X. Gao, L. Yuan, M. Ahmad, J. G. Bian, G. M. Chen, H. S. Chen, M. Chen, Y. Chen, C. H. Jiang, D. Leggat, H. Liao, Z. Liu, F. Romeo, S. M. Shaheen, A. Spiezia, J. Tao, C. Wang, Z. Wang, E. Yazgan, H. Zhang, S. Zhang, J. Zhao, Y. Ban, G. Chen, J. Li, Q. Li, S. Liu, Y. Mao, S. J. Qian, D. Wang, Z. Xu, F. Zhang, Y. Wang, C. Avila, A. Cabrera, L. F. Chaparro Sierra, C. Florez, C. F. González Hernández, J. D. Ruiz Alvarez, M. A. Segura Delgado, B. Courbon, N. Godinovic, D. Lelas, I. Puljak, P. M. Ribeiro Cipriano, T. Sculac, Z. Antunovic, M. Kovac, V. Brigljevic, D. Ferencek, K. Kadija, B. Mesic, A. Starodumov, T. Susa, M. W. Ather, A. Attikis, G. Mavromanolakis, J. Mousa, C. Nicolaou, F. Ptochos, P. A. Razis, H. Rykaczewski, M. Finger, M. Finger, E. Carrera Jarrin, Y. Assran, S. Elgammal, A. Mahrous, R. K. Dewanjee, M. Kadastik, L. Perrini, M. Raidal, A. Tiko, C. Veelken, P. Eerola, H. Kirschenmann, J. Pekkanen, M. Voutilainen, J. Havukainen, J. K. Heikkilä, T. Järvinen, V. Karimäki, R. Kinnunen, T. Lampén, K. Lassila-Perini, S. Laurila, S. Lehti, T. Lindén, P. Luukka, H. Siikonen, E. Tuominen, J. Tuominiemi, T. Tuuva, M. Besancon, F. Couderc, M. Dejardin, D. Denegri, J. L. Faure, F. Ferri, S. Ganjour, S. Ghosh, P. Gras, G. Hamel de Monchenault, P. Jarry, I. Kucher, C. Leloup, E. Locci, M. Machet, J. Malcles, G. Negro, J. Rander, A. Rosowsky, M. Ö. Sahin, M. Titov, A. Abdulsalam, C. Amendola, I. Antropov, S. Baffioni, F. Beaudette, P. Busson, L. Cadamuro, C. Charlot, R. Granier de Cassagnac, M. Jo, S. Lisniak, A. Lobanov, J. Martin Blanco, M. Nguyen, C. Ochando, G. Ortona, P. Paganini, P. Pigard, R. Salerno, J. B. Sauvan, Y. Sirois, A. G. Stahl Leiton, T. Strebler, Y. Yilmaz, A. Zabi, A. Zghiche, J.-L. Agram, J. Andrea, D. Bloch, J.-M. Brom, M. Buttignol, E. C. Chabert, N. Chanon, C. Collard, E. Conte, X. Coubez, J.-C. Fontaine, D. Gelé, U. Goerlach, M. Jansová, A.-C. Le Bihan, N. Tonon, P. Van Hove, S. Gadrat, S. Beauceron, C. Bernet, G. Boudoul, R. Chierici, D. Contardo, P. Depasse, H. El Mamouni, J. Fay, L. Finco, S. Gascon, M. Gouzevitch, G. Grenier, B. Ille, F. Lagarde, I. B. Laktineh, M. Lethuillier, L. Mirabito, A. L. Pequegnot, S. Perries, A. Popov, V. Sordini, M. Vander Donckt, S. Viret, A. Khvedelidze, D. Lomidze, C. Autermann, L. Feld, M. K. Kiesel, K. Klein, M. Lipinski, M. Preuten, C. Schomakers, J. Schulz, M. Teroerde, V. Zhukov, A. Albert, E. Dietz-Laursonn, D. Duchardt, M. Endres, M. Erdmann, S. Erdweg, T. Esch, R. Fischer, A. Güth, M. Hamer, T. Hebbeker, C. Heidemann, K. Hoepfner, S. Knutzen, M. Merschmeyer, A. Meyer, P. Millet, S. Mukherjee, T. Pook, M. Radziej, H. Reithler, M. Rieger, F. Scheuch, D. Teyssier, S. Thüer, G. Flügge, B. Kargoll, T. Kress, A. Künsken, T. Müller, A. Nehrkorn, A. Nowack, C. Pistone, O. Pooth, A. Stahl, M. Aldaya Martin, T. Arndt, C. Asawatangtrakuldee, K. Beernaert, O. Behnke, U. Behrens, A. Bermúdez Martínez, A. A. Bin Anuar, K. Borras, V. Botta, A. Campbell, P. Connor, C. Contreras-Campana, F. Costanza, C. Diez Pardos, G. Eckerlin, D. Eckstein, T. Eichhorn, E. Eren, E. Gallo, J. Garay Garcia, A. Geiser, J. M. Grados Luyando, A. Grohsjean, P. Gunnellini, M. Guthoff, A. Harb, J. Hauk, M. Hempel, H. Jung, A. Kasem, M. Kasemann, J. Keaveney, C. Kleinwort, I. Korol, D. Krücker, W. Lange, A. Lelek, T. Lenz, J. Leonard, K. Lipka, W. Lohmann, R. Mankel, I.-A. Melzer-Pellmann, A. B. Meyer, G. Mittag, J. Mnich, A. Mussgiller, E. Ntomari, D. Pitzl, A. Raspereza, M. Savitskyi, P. Saxena, R. Shevchenko, N. Stefaniuk, G. P. Van Onsem, R. Walsh, Y. Wen, K. Wichmann, C. Wissing, O. Zenaiev, R. Aggleton, S. Bein, V. Blobel, M. Centis Vignali, T. Dreyer, E. Garutti, D. Gonzalez, J. Haller, A. Hinzmann, M. Hoffmann, A. Karavdina, R. Klanner, R. Kogler, N. Kovalchuk, S. Kurz, T. Lapsien, D. Marconi, M. Meyer, M. Niedziela, D. Nowatschin, F. Pantaleo, T. Peiffer, A. Perieanu, C. Scharf, P. Schleper, A. Schmidt, S. Schumann, J. Schwandt, J. Sonneveld, H. Stadie, G. Steinbrück, F. M. Stober, M. Stöver, H. Tholen, D. Troendle, E. Usai, A. Vanhoefer, B. Vormwald, M. Akbiyik, C. Barth, M. Baselga, S. Baur, E. Butz, R. Caspart, T. Chwalek, F. Colombo, W. De Boer, A. Dierlamm, N. Faltermann, B. Freund, R. Friese, M. Giffels, M. A. Harrendorf, F. Hartmann, S. M. Heindl, U. Husemann, F. Kassel, S. Kudella, H. Mildner, M. U. Mozer, Th. Müller, M. Plagge, G. Quast, K. Rabbertz, M. Schröder, I. Shvetsov, G. Sieber, H. J. Simonis, R. Ulrich, S. Wayand, M. Weber, T. Weiler, S. Williamson, C. Wöhrmann, R. Wolf, G. Anagnostou, G. Daskalakis, T. Geralis, A. Kyriakis, D. Loukas, I. Topsis-Giotis, G. Karathanasis, S. Kesisoglou, A. Panagiotou, N. Saoulidou, K. Kousouris, I. Evangelou, C. Foudas, P. Gianneios, P. Katsoulis, P. Kokkas, S. Mallios, N. Manthos, I. Papadopoulos, E. Paradas, J. Strologas, F. A. Triantis, D. Tsitsonis, M. Csanad, N. Filipovic, G. Pasztor, O. Surányi, G. I. Veres, G. Bencze, C. Hajdu, D. Horvath, Á. Hunyadi, F. Sikler, V. Veszpremi, N. Beni, S. Czellar, J. Karancsi, A. Makovec, J. Molnar, Z. Szillasi, M. Bartók, P. Raics, Z. L. Trocsanyi, B. Ujvari, S. Choudhury, J. R. Komaragiri, S. Bahinipati, S. Bhowmik, P. Mal, K. Mandal, A. Nayak, D. K. Sahoo, N. Sahoo, S. K. Swain, S. Bansal, S. B. Beri, V. Bhatnagar, R. Chawla, N. Dhingra, A. Kaur, M. Kaur, S. Kaur, R. Kumar, P. Kumari, A. Mehta, J. B. Singh, G. Walia, Ashok Kumar, Aashaq Shah, A. Bhardwaj, S. Chauhan, B. C. Choudhary, R. B. Garg, S. Keshri, A. Kumar, S. Malhotra, M. Naimuddin, K. Ranjan, R. Sharma, R. Bhardwaj, R. Bhattacharya, S. Bhattacharya, U. Bhawandeep, S. Dey, S. Dutt, S. Dutta, S. Ghosh, N. Majumdar, A. Modak, K. Mondal, S. Mukhopadhyay, S. Nandan, A. Purohit, A. Roy, S. Roy Chowdhury, S. Sarkar, M. Sharan, S. Thakur, P. K. Behera, R. Chudasama, D. Dutta, V. Jha, V. Kumar, A. K. Mohanty, P. K. Netrakanti, L. M. Pant, P. Shukla, A. Topkar, T. Aziz, S. Dugad, B. Mahakud, S. Mitra, G. B. Mohanty, N. Sur, B. Sutar, S. Banerjee, S. Bhattacharya, S. Chatterjee, P. Das, M. Guchait, Sa. Jain, S. Kumar, M. Maity, G. Majumder, K. Mazumdar, T. Sarkar, N. Wickramage, S. Chauhan, S. Dube, V. Hegde, A. Kapoor, K. Kothekar, S. Pandey, A. Rane, S. Sharma, S. Chenarani, E. Eskandari Tadavani, S. M. Etesami, M. Khakzad, M. Mohammadi Najafabadi, M. Naseri, S. Paktinat Mehdiabadi, F. Rezaei Hosseinabadi, B. Safarzadeh, M. Zeinali, M. Felcini, M. Grunewald, M. Abbrescia, C. Calabria, A. Colaleo, D. Creanza, L. Cristella, N. De Filippis, M. De Palma, F. Errico, L. Fiore, G. Iaselli, S. Lezki, G. Maggi, M. Maggi, G. Miniello, S. My, S. Nuzzo, A. Pompili, G. Pugliese, R. Radogna, A. Ranieri, G. Selvaggi, A. Sharma, L. Silvestris, R. Venditti, P. Verwilligen, G. Abbiendi, C. Battilana, D. Bonacorsi, L. Borgonovi, S. Braibant-Giacomelli, R. Campanini, P. Capiluppi, A. Castro, F. R. Cavallo, S. S. Chhibra, G. Codispoti, M. Cuffiani, G. M. Dallavalle, F. Fabbri, A. Fanfani, D. Fasanella, P. Giacomelli, C. Grandi, L. Guiducci, S. Marcellini, G. Masetti, A. Montanari, F. L. Navarria, A. Perrotta, A. M. Rossi, T. Rovelli, G. P. Siroli, N. Tosi, S. Albergo, S. Costa, A. Di Mattia, F. Giordano, R. Potenza, A. Tricomi, C. Tuve, G. Barbagli, K. Chatterjee, V. Ciulli, C. Civinini, R. D’Alessandro, E. Focardi, P. Lenzi, M. Meschini, S. Paoletti, L. Russo, G. Sguazzoni, D. Strom, L. Viliani, L. Benussi, S. Bianco, F. Fabbri, D. Piccolo, F. Primavera, V. Calvelli, F. Ferro, F. Ravera, E. Robutti, S. Tosi, A. Benaglia, A. Beschi, L. Brianza, F. Brivio, V. Ciriolo, M. E. Dinardo, S. Fiorendi, S. Gennai, A. Ghezzi, P. Govoni, M. Malberti, S. Malvezzi, R. A. Manzoni, D. Menasce, L. Moroni, M. Paganoni, K. Pauwels, D. Pedrini, S. Pigazzini, S. Ragazzi, T. Tabarelli de Fatis, S. Buontempo, N. Cavallo, S. Di Guida, F. Fabozzi, F. Fienga, A. O. M. Iorio, W. A. Khan, L. Lista, S. Meola, P. Paolucci, C. Sciacca, F. Thyssen, P. Azzi, N. Bacchetta, L. Benato, D. Bisello, A. Boletti, A. Carvalho Antunes De Oliveira, P. Checchia, M. Dall’Osso, P. De Castro Manzano, T. Dorigo, U. Dosselli, F. Gasparini, U. Gasparini, A. Gozzelino, S. Lacaprara, P. Lujan, M. Margoni, A. T. Meneguzzo, N. Pozzobon, P. Ronchese, R. Rossin, F. Simonetto, E. Torassa, M. Zanetti, P. Zotto, G. Zumerle, A. Braghieri, A. Magnani, P. Montagna, S. P. Ratti, V. Re, M. Ressegotti, C. Riccardi, P. Salvini, I. Vai, P. Vitulo, L. Alunni Solestizi, M. Biasini, G. M. Bilei, C. Cecchi, D. Ciangottini, L. Fanò, R. Leonardi, E. Manoni, G. Mantovani, V. Mariani, M. Menichelli, A. Rossi, A. Santocchia, D. Spiga, K. Androsov, P. Azzurri, G. Bagliesi, T. Boccali, L. Borrello, R. Castaldi, M. A. Ciocci, R. Dell’Orso, G. Fedi, L. Giannini, A. Giassi, M. T. Grippo, F. Ligabue, T. Lomtadze, E. Manca, G. Mandorli, A. Messineo, F. Palla, A. Rizzi, A. Savoy-Navarro, P. Spagnolo, R. Tenchini, G. Tonelli, A. Venturi, P. G. Verdini, L. Barone, F. Cavallari, M. Cipriani, N. Daci, D. Del Re, E. Di Marco, M. Diemoz, S. Gelli, E. Longo, F. Margaroli, B. Marzocchi, P. Meridiani, G. Organtini, R. Paramatti, F. Preiato, S. Rahatlou, C. Rovelli, F. Santanastasio, N. Amapane, R. Arcidiacono, S. Argiro, M. Arneodo, N. Bartosik, R. Bellan, C. Biino, N. Cartiglia, F. Cenna, M. Costa, R. Covarelli, A. Degano, N. Demaria, B. Kiani, C. Mariotti, S. Maselli, E. Migliore, V. Monaco, E. Monteil, M. Monteno, M. M. Obertino, L. Pacher, N. Pastrone, M. Pelliccioni, G. L. Pinna Angioni, A. Romero, M. Ruspa, R. Sacchi, K. Shchelina, V. Sola, A. Solano, A. Staiano, P. Traczyk, S. Belforte, M. Casarsa, F. Cossutti, G. Della Ricca, A. Zanetti, D. H. Kim, G. N. Kim, M. S. Kim, J. Lee, S. Lee, S. W. Lee, C. S. Moon, Y. D. Oh, S. Sekmen, D. C. Son, Y. C. Yang, A. Lee, H. Kim, D. H. Moon, G. Oh, J. A. Brochero Cifuentes, J. Goh, T. J. Kim, S. Cho, S. Choi, Y. Go, D. Gyun, S. Ha, B. Hong, Y. Jo, Y. Kim, K. Lee, K. S. Lee, S. Lee, J. Lim, S. K. Park, Y. Roh, J. Almond, J. Kim, J. S. Kim, H. Lee, K. Lee, K. Nam, S. B. Oh, B. C. Radburn-Smith, S. h. Seo, U. K. Yang, H. D. Yoo, G. B. Yu, H. Kim, J. H. Kim, J. S. H. Lee, I. C. Park, Y. Choi, C. Hwang, J. Lee, I. Yu, V. Dudenas, A. Juodagalvis, J. Vaitkus, I. Ahmed, Z. A. Ibrahim, M. A. B. Md Ali, F. Mohamad Idris, W. A. T. Wan Abdullah, M. N. Yusli, Z. Zolkapli, R. Reyes-Almanza, G. Ramirez-Sanchez, M. C. Duran-Osuna, H. Castilla-Valdez, E. De La Cruz-Burelo, I. Heredia-De La Cruz, R. I. Rabadan-Trejo, R. Lopez-Fernandez, J. Mejia Guisao, A. Sanchez-Hernandez, S. Carrillo Moreno, C. Oropeza Barrera, F. Vazquez Valencia, J. Eysermans, I. Pedraza, H. A. Salazar Ibarguen, C. Uribe Estrada, A. Morelos Pineda, D. Krofcheck, P. H. Butler, A. Ahmad, M. Ahmad, Q. Hassan, H. R. Hoorani, A. Saddique, M. A. Shah, M. Shoaib, M. Waqas, H. Bialkowska, M. Bluj, B. Boimska, T. Frueboes, M. Górski, M. Kazana, K. Nawrocki, M. Szleper, P. Zalewski, K. Bunkowski, A. Byszuk, K. Doroba, A. Kalinowski, M. Konecki, J. Krolikowski, M. Misiura, M. Olszewski, A. Pyskir, M. Walczak, P. Bargassa, C. Beirão Da Cruz E. Silva, A. Di Francesco, P. Faccioli, B. Galinhas, M. Gallinaro, J. Hollar, N. Leonardo, L. Lloret Iglesias, M. V. Nemallapudi, J. Seixas, G. Strong, O. Toldaiev, D. Vadruccio, J. Varela, S. Afanasiev, A. Golunov, I. Golutvin, N. Gorbounov, A. Kamenev, V. Karjavin, A. Lanev, A. Malakhov, V. Matveev, V. Palichik, V. Perelygin, M. Savina, S. Shmatov, S. Shulha, N. Skatchkov, V. Smirnov, N. Voytishin, A. Zarubin, Y. Ivanov, V. Kim, E. Kuznetsova, P. Levchenko, V. Murzin, V. Oreshkin, I. Smirnov, D. Sosnov, V. Sulimov, L. Uvarov, S. Vavilov, A. Vorobyev, Yu. Andreev, A. Dermenev, S. Gninenko, N. Golubev, A. Karneyeu, M. Kirsanov, N. Krasnikov, A. Pashenkov, D. Tlisov, A. Toropin, V. Epshteyn, V. Gavrilov, N. Lychkovskaya, V. Popov, I. Pozdnyakov, G. Safronov, A. Spiridonov, A. Stepennov, M. Toms, E. Vlasov, A. Zhokin, T. Aushev, A. Bylinkin, M. Chadeeva, P. Parygin, D. Philippov, S. Polikarpov, E. Popova, V. Rusinov, E. Zhemchugov, V. Andreev, M. Azarkin, I. Dremin, M. Kirakosyan, A. Terkulov, A. Baskakov, A. Belyaev, E. Boos, M. Dubinin, L. Dudko, A. Ershov, A. Gribushin, V. Klyukhin, O. Kodolova, I. Lokhtin, I. Miagkov, S. Obraztsov, S. Petrushanko, V. Savrin, A. Snigirev, V. Blinov, Y. Skovpen, D. Shtol, I. Azhgirey, I. Bayshev, S. Bitioukov, D. Elumakhov, A. Godizov, V. Kachanov, A. Kalinin, D. Konstantinov, P. Mandrik, V. Petrov, R. Ryutin, A. Sobol, S. Troshin, N. Tyurin, A. Uzunian, A. Volkov, P. Adzic, P. Cirkovic, D. Devetak, M. Dordevic, J. Milosevic, V. Rekovic, J. Alcaraz Maestre, I. Bachiller, M. Barrio Luna, M. Cerrada, N. Colino, B. De La Cruz, A. Delgado Peris, C. Fernandez Bedoya, J. P. Fernández Ramos, J. Flix, M. C. Fouz, O. Gonzalez Lopez, S. Goy Lopez, J. M. Hernandez, M. I. Josa, D. Moran, A. Pérez-Calero Yzquierdo, J. Puerta Pelayo, A. Quintario Olmeda, I. Redondo, L. Romero, M. S. Soares, A. Álvarez Fernández, C. Albajar, J. F. de Trocóniz, M. Missiroli, J. Cuevas, C. Erice, J. Fernandez Menendez, I. Gonzalez Caballero, J. R. González Fernández, E. Palencia Cortezon, S. Sanchez Cruz, P. Vischia, J. M. Vizan Garcia, I. J. Cabrillo, A. Calderon, B. Chazin Quero, E. Curras, J. Duarte Campderros, M. Fernandez, J. Garcia-Ferrero, G. Gomez, A. Lopez Virto, J. Marco, C. Martinez Rivero, P. Martinez Ruiz del Arbol, F. Matorras, J. Piedra Gomez, T. Rodrigo, A. Ruiz-Jimeno, L. Scodellaro, N. Trevisani, I. Vila, R. Vilar Cortabitarte, D. Abbaneo, B. Akgun, E. Auffray, P. Baillon, A. H. Ball, D. Barney, J. Bendavid, M. Bianco, P. Bloch, A. Bocci, C. Botta, T. Camporesi, R. Castello, M. Cepeda, G. Cerminara, E. Chapon, Y. Chen, D. d’Enterria, A. Dabrowski, V. Daponte, A. David, M. De Gruttola, A. De Roeck, N. Deelen, M. Dobson, T. du Pree, M. Dünser, N. Dupont, A. Elliott-Peisert, P. Everaerts, F. Fallavollita, G. Franzoni, J. Fulcher, W. Funk, D. Gigi, A. Gilbert, K. Gill, F. Glege, D. Gulhan, P. Harris, J. Hegeman, V. Innocente, A. Jafari, P. Janot, O. Karacheban, J. Kieseler, V. Knünz, A. Kornmayer, M. J. Kortelainen, M. Krammer, C. Lange, P. Lecoq, C. Lourenço, M. T. Lucchini, L. Malgeri, M. Mannelli, A. Martelli, F. Meijers, J. A. Merlin, S. Mersi, E. Meschi, P. Milenovic, F. Moortgat, M. Mulders, H. Neugebauer, J. Ngadiuba, S. Orfanelli, L. Orsini, L. Pape, E. Perez, M. Peruzzi, A. Petrilli, G. Petrucciani, A. Pfeiffer, M. Pierini, D. Rabady, A. Racz, T. Reis, G. Rolandi, M. Rovere, H. Sakulin, C. Schäfer, C. Schwick, M. Seidel, M. Selvaggi, A. Sharma, P. Silva, P. Sphicas, A. Stakia, J. Steggemann, M. Stoye, M. Tosi, D. Treille, A. Triossi, A. Tsirou, V. Veckalns, M. Verweij, W. D. Zeuner, W. Bertl, L. Caminada, K. Deiters, W. Erdmann, R. Horisberger, Q. Ingram, H. C. Kaestli, D. Kotlinski, U. Langenegger, T. Rohe, S. A. Wiederkehr, M. Backhaus, L. Bäni, P. Berger, L. Bianchini, B. Casal, G. Dissertori, M. Dittmar, M. Donegà, C. Dorfer, C. Grab, C. Heidegger, D. Hits, J. Hoss, G. Kasieczka, T. Klijnsma, W. Lustermann, B. Mangano, M. Marionneau, M. T. Meinhard, D. Meister, F. Micheli, P. Musella, F. Nessi-Tedaldi, F. Pandolfi, J. Pata, F. Pauss, G. Perrin, L. Perrozzi, M. Quittnat, M. Reichmann, D. A. Sanz Becerra, M. Schönenberger, L. Shchutska, V. R. Tavolaro, K. Theofilatos, M. L. Vesterbacka Olsson, R. Wallny, D. H. Zhu, T. K. Aarrestad, C. Amsler, M. F. Canelli, A. De Cosa, R. Del Burgo, S. Donato, C. Galloni, T. Hreus, B. Kilminster, D. Pinna, G. Rauco, P. Robmann, D. Salerno, K. Schweiger, C. Seitz, Y. Takahashi, A. Zucchetta, V. Candelise, Y. H. Chang, K. y. Cheng, T. H. Doan, Sh. Jain, R. Khurana, C. M. Kuo, W. Lin, A. Pozdnyakov, S. S. Yu, Arun Kumar, P. Chang, Y. Chao, K. F. Chen, P. H. Chen, F. Fiori, W.-S. Hou, Y. Hsiung, Y. F. Liu, R.-S. Lu, E. Paganis, A. Psallidas, A. Steen, J. f. Tsai, B. Asavapibhop, K. Kovitanggoon, G. Singh, N. Srimanobhas, A. Bat, F. Boran, S. Cerci, S. Damarseckin, Z. S. Demiroglu, C. Dozen, I. Dumanoglu, S. Girgis, G. Gokbulut, Y. Guler, I. Hos, E. E. Kangal, O. Kara, A. Kayis Topaksu, U. Kiminsu, M. Oglakci, G. Onengut, K. Ozdemir, D. Sunar Cerci, B. Tali, U. G. Tok, S. Turkcapar, I. S. Zorbakir, C. Zorbilmez, G. Karapinar, K. Ocalan, M. Yalvac, M. Zeyrek, E. Gülmez, M. Kaya, O. Kaya, S. Tekten, E. A. Yetkin, M. N. Agaras, S. Atay, A. Cakir, K. Cankocak, I. Köseoglu, B. Grynyov, L. Levchuk, F. Ball, L. Beck, J. J. Brooke, D. Burns, E. Clement, D. Cussans, O. Davignon, H. Flacher, J. Goldstein, G. P. Heath, H. F. Heath, L. Kreczko, D. M. Newbold, S. Paramesvaran, T. Sakuma, S. Seif El Nasr-storey, D. Smith, V. J. Smith, K. W. Bell, A. Belyaev, C. Brew, R. M. Brown, L. Calligaris, D. Cieri, D. J. A. Cockerill, J. A. Coughlan, K. Harder, S. Harper, J. Linacre, E. Olaiya, D. Petyt, C. H. Shepherd-Themistocleous, A. Thea, I. R. Tomalin, T. Williams, G. Auzinger, R. Bainbridge, J. Borg, S. Breeze, O. Buchmuller, A. Bundock, S. Casasso, M. Citron, D. Colling, L. Corpe, P. Dauncey, G. Davies, A. De Wit, M. Della Negra, R. Di Maria, A. Elwood, Y. Haddad, G. Hall, G. Iles, T. James, R. Lane, C. Laner, L. Lyons, A.-M. Magnan, S. Malik, L. Mastrolorenzo, T. Matsushita, J. Nash, A. Nikitenko, V. Palladino, M. Pesaresi, D. M. Raymond, A. Richards, A. Rose, E. Scott, C. Seez, A. Shtipliyski, S. Summers, A. Tapper, K. Uchida, M. Vazquez Acosta, T. Virdee, N. Wardle, D. Winterbottom, J. Wright, S. C. Zenz, J. E. Cole, P. R. Hobson, A. Khan, P. Kyberd, I. D. Reid, L. Teodorescu, S. Zahid, A. Borzou, K. Call, J. Dittmann, K. Hatakeyama, H. Liu, N. Pastika, C. Smith, R. Bartek, A. Dominguez, A. Buccilli, S. I. Cooper, C. Henderson, P. Rumerio, C. West, D. Arcaro, A. Avetisyan, T. Bose, D. Gastler, D. Rankin, C. Richardson, J. Rohlf, L. Sulak, D. Zou, G. Benelli, D. Cutts, A. Garabedian, M. Hadley, J. Hakala, U. Heintz, J. M. Hogan, K. H. M. Kwok, E. Laird, G. Landsberg, J. Lee, Z. Mao, M. Narain, J. Pazzini, S. Piperov, S. Sagir, R. Syarif, D. Yu, R. Band, C. Brainerd, R. Breedon, D. Burns, M. Calderon De La Barca Sanchez, M. Chertok, J. Conway, R. Conway, P. T. Cox, R. Erbacher, C. Flores, G. Funk, W. Ko, R. Lander, C. Mclean, M. Mulhearn, D. Pellett, J. Pilot, S. Shalhout, M. Shi, J. Smith, D. Stolp, K. Tos, M. Tripathi, Z. Wang, M. Bachtis, C. Bravo, R. Cousins, A. Dasgupta, A. Florent, J. Hauser, M. Ignatenko, N. Mccoll, S. Regnard, D. Saltzberg, C. Schnaible, V. Valuev, E. Bouvier, K. Burt, R. Clare, J. Ellison, J. W. Gary, S. M. A. Ghiasi Shirazi, G. Hanson, J. Heilman, G. Karapostoli, E. Kennedy, F. Lacroix, O. R. Long, M. Olmedo Negrete, M. I. Paneva, W. Si, L. Wang, H. Wei, S. Wimpenny, B. R. Yates, J. G. Branson, S. Cittolin, M. Derdzinski, R. Gerosa, D. Gilbert, B. Hashemi, A. Holzner, D. Klein, G. Kole, V. Krutelyov, J. Letts, M. Masciovecchio, D. Olivito, S. Padhi, M. Pieri, M. Sani, V. Sharma, M. Tadel, A. Vartak, S. Wasserbaech, J. Wood, F. Würthwein, A. Yagil, G. Zevi Della Porta, N. Amin, R. Bhandari, J. Bradmiller-Feld, C. Campagnari, A. Dishaw, V. Dutta, M. Franco Sevilla, L. Gouskos, R. Heller, J. Incandela, A. Ovcharova, H. Qu, J. Richman, D. Stuart, I. Suarez, J. Yoo, D. Anderson, A. Bornheim, J. M. Lawhorn, H. B. Newman, T. Q. Nguyen, C. Pena, M. Spiropulu, J. R. Vlimant, S. Xie, Z. Zhang, R. Y. Zhu, M. B. Andrews, T. Ferguson, T. Mudholkar, M. Paulini, J. Russ, M. Sun, H. Vogel, I. Vorobiev, M. Weinberg, J. P. Cumalat, W. T. Ford, F. Jensen, A. Johnson, M. Krohn, S. Leontsinis, T. Mulholland, K. Stenson, S. R. Wagner, J. Alexander, J. Chaves, J. Chu, S. Dittmer, K. Mcdermott, N. Mirman, J. R. Patterson, D. Quach, A. Rinkevicius, A. Ryd, L. Skinnari, L. Soffi, S. M. Tan, Z. Tao, J. Thom, J. Tucker, P. Wittich, M. Zientek, S. Abdullin, M. Albrow, M. Alyari, G. Apollinari, A. Apresyan, A. Apyan, S. Banerjee, L. A. T. Bauerdick, A. Beretvas, J. Berryhill, P. C. Bhat, G. Bolla, K. Burkett, J. N. Butler, A. Canepa, G. B. Cerati, H. W. K. Cheung, F. Chlebana, M. Cremonesi, J. Duarte, V. D. Elvira, J. Freeman, Z. Gecse, E. Gottschalk, L. Gray, D. Green, S. Grünendahl, O. Gutsche, R. M. Harris, S. Hasegawa, J. Hirschauer, Z. Hu, B. Jayatilaka, S. Jindariani, M. Johnson, U. Joshi, B. Klima, B. Kreis, S. Lammel, D. Lincoln, R. Lipton, M. Liu, T. Liu, R. Lopes De Sá, J. Lykken, K. Maeshima, N. Magini, J. M. Marraffino, D. Mason, P. McBride, P. Merkel, S. Mrenna, S. Nahn, V. O’Dell, K. Pedro, O. Prokofyev, G. Rakness, L. Ristori, B. Schneider, E. Sexton-Kennedy, A. Soha, W. J. Spalding, L. Spiegel, S. Stoynev, J. Strait, N. Strobbe, L. Taylor, S. Tkaczyk, N. V. Tran, L. Uplegger, E. W. Vaandering, C. Vernieri, M. Verzocchi, R. Vidal, M. Wang, H. A. Weber, A. Whitbeck, D. Acosta, P. Avery, P. Bortignon, D. Bourilkov, A. Brinkerhoff, A. Carnes, M. Carver, D. Curry, R. D. Field, I. K. Furic, S. V. Gleyzer, B. M. Joshi, J. Konigsberg, A. Korytov, K. Kotov, P. Ma, K. Matchev, H. Mei, G. Mitselmakher, K. Shi, D. Sperka, N. Terentyev, L. Thomas, J. Wang, S. Wang, J. Yelton, Y. R. Joshi, S. Linn, P. Markowitz, J. L. Rodriguez, A. Ackert, T. Adams, A. Askew, S. Hagopian, V. Hagopian, K. F. Johnson, T. Kolberg, G. Martinez, T. Perry, H. Prosper, A. Saha, A. Santra, V. Sharma, R. Yohay, M. M. Baarmand, V. Bhopatkar, S. Colafranceschi, M. Hohlmann, D. Noonan, T. Roy, F. Yumiceva, M. R. Adams, L. Apanasevich, D. Berry, R. R. Betts, R. Cavanaugh, X. Chen, O. Evdokimov, C. E. Gerber, D. A. Hangal, D. J. Hofman, K. Jung, J. Kamin, I. D. Sandoval Gonzalez, M. B. Tonjes, H. Trauger, N. Varelas, H. Wang, Z. Wu, J. Zhang, B. Bilki, W. Clarida, K. Dilsiz, S. Durgut, R. P. Gandrajula, M. Haytmyradov, V. Khristenko, J.-P. Merlo, H. Mermerkaya, A. Mestvirishvili, A. Moeller, J. Nachtman, H. Ogul, Y. Onel, F. Ozok, A. Penzo, C. Snyder, E. Tiras, J. Wetzel, K. Yi, B. Blumenfeld, A. Cocoros, N. Eminizer, D. Fehling, L. Feng, A. V. Gritsan, P. Maksimovic, J. Roskes, U. Sarica, M. Swartz, M. Xiao, C. You, A. Al-bataineh, P. Baringer, A. Bean, S. Boren, J. Bowen, J. Castle, S. Khalil, A. Kropivnitskaya, D. Majumder, W. Mcbrayer, M. Murray, C. Rogan, C. Royon, S. Sanders, E. Schmitz, J. D. Tapia Takaki, Q. Wang, A. Ivanov, K. Kaadze, Y. Maravin, A. Mohammadi, L. K. Saini, N. Skhirtladze, F. Rebassoo, D. Wright, C. Anelli, A. Baden, O. Baron, A. Belloni, S. C. Eno, Y. Feng, C. Ferraioli, N. J. Hadley, S. Jabeen, G. Y. Jeng, R. G. Kellogg, J. Kunkle, A. C. Mignerey, F. Ricci-Tam, Y. H. Shin, A. Skuja, S. C. Tonwar, D. Abercrombie, B. Allen, V. Azzolini, R. Barbieri, A. Baty, R. Bi, S. Brandt, W. Busza, I. A. Cali, M. D’Alfonso, Z. Demiragli, G. Gomez Ceballos, M. Goncharov, D. Hsu, M. Hu, Y. Iiyama, G. M. Innocenti, M. Klute, D. Kovalskyi, Y.-J. Lee, A. Levin, P. D. Luckey, B. Maier, A. C. Marini, C. Mcginn, C. Mironov, S. Narayanan, X. Niu, C. Paus, C. Roland, G. Roland, J. Salfeld-Nebgen, G. S. F. Stephans, K. Tatar, D. Velicanu, J. Wang, T. W. Wang, B. Wyslouch, A. C. Benvenuti, R. M. Chatterjee, A. Evans, P. Hansen, J. Hiltbrand, S. Kalafut, Y. Kubota, Z. Lesko, J. Mans, S. Nourbakhsh, N. Ruckstuhl, R. Rusack, J. Turkewitz, M. A. Wadud, J. G. Acosta, S. Oliveros, E. Avdeeva, K. Bloom, D. R. Claes, C. Fangmeier, F. Golf, R. Gonzalez Suarez, R. Kamalieddin, I. Kravchenko, J. Monroy, J. E. Siado, G. R. Snow, B. Stieger, J. Dolen, A. Godshalk, C. Harrington, I. Iashvili, D. Nguyen, A. Parker, S. Rappoccio, B. Roozbahani, G. Alverson, E. Barberis, C. Freer, A. Hortiangtham, A. Massironi, D. M. Morse, T. Orimoto, R. Teixeira De Lima, D. Trocino, T. Wamorkar, B. Wang, A. Wisecarver, D. Wood, S. Bhattacharya, O. Charaf, K. A. Hahn, N. Mucia, N. Odell, M. H. Schmitt, K. Sung, M. Trovato, M. Velasco, R. Bucci, N. Dev, M. Hildreth, K. Hurtado Anampa, C. Jessop, D. J. Karmgard, N. Kellams, K. Lannon, W. Li, N. Loukas, N. Marinelli, F. Meng, C. Mueller, Y. Musienko, M. Planer, A. Reinsvold, R. Ruchti, P. Siddireddy, G. Smith, S. Taroni, M. Wayne, A. Wightman, M. Wolf, A. Woodard, J. Alimena, L. Antonelli, B. Bylsma, L. S. Durkin, S. Flowers, B. Francis, A. Hart, C. Hill, W. Ji, B. Liu, W. Luo, B. L. Winer, H. W. Wulsin, S. Cooperstein, O. Driga, P. Elmer, J. Hardenbrook, P. Hebda, S. Higginbotham, A. Kalogeropoulos, D. Lange, J. Luo, D. Marlow, K. Mei, I. Ojalvo, J. Olsen, C. Palmer, P. Piroué, D. Stickland, C. Tully, S. Malik, S. Norberg, A. Barker, V. E. Barnes, S. Das, S. Folgueras, L. Gutay, M. K. Jha, M. Jones, A. W. Jung, A. Khatiwada, D. H. Miller, N. Neumeister, C. C. Peng, H. Qiu, J. F. Schulte, J. Sun, F. Wang, R. Xiao, W. Xie, T. Cheng, N. Parashar, J. Stupak, Z. Chen, K. M. Ecklund, S. Freed, F. J. M. Geurts, M. Guilbaud, M. Kilpatrick, W. Li, B. Michlin, B. P. Padley, J. Roberts, J. Rorie, W. Shi, Z. Tu, J. Zabel, A. Zhang, A. Bodek, P. de Barbaro, R. Demina, Y. t. Duh, T. Ferbel, M. Galanti, A. Garcia-Bellido, J. Han, O. Hindrichs, A. Khukhunaishvili, K. H. Lo, P. Tan, M. Verzetti, R. Ciesielski, K. Goulianos, C. Mesropian, A. Agapitos, J. P. Chou, Y. Gershtein, T. A. Gómez Espinosa, E. Halkiadakis, M. Heindl, E. Hughes, S. Kaplan, R. Kunnawalkam Elayavalli, S. Kyriacou, A. Lath, R. Montalvo, K. Nash, M. Osherson, H. Saka, S. Salur, S. Schnetzer, D. Sheffield, S. Somalwar, R. Stone, S. Thomas, P. Thomassen, M. Walker, A. G. Delannoy, J. Heideman, G. Riley, K. Rose, S. Spanier, K. Thapa, O. Bouhali, A. Castaneda Hernandez, A. Celik, M. Dalchenko, M. De Mattia, A. Delgado, S. Dildick, R. Eusebi, J. Gilmore, T. Huang, T. Kamon, R. Mueller, Y. Pakhotin, R. Patel, A. Perloff, L. Perniè, D. Rathjens, A. Safonov, A. Tatarinov, K. A. Ulmer, N. Akchurin, J. Damgov, F. De Guio, P. R. Dudero, J. Faulkner, E. Gurpinar, S. Kunori, K. Lamichhane, S. W. Lee, T. Libeiro, T. Mengke, S. Muthumuni, T. Peltola, S. Undleeb, I. Volobouev, Z. Wang, S. Greene, A. Gurrola, R. Janjam, W. Johns, C. Maguire, A. Melo, H. Ni, K. Padeken, P. Sheldon, S. Tuo, J. Velkovska, Q. Xu, M. W. Arenton, P. Barria, B. Cox, R. Hirosky, M. Joyce, A. Ledovskoy, H. Li, C. Neu, T. Sinthuprasith, Y. Wang, E. Wolfe, F. Xia, R. Harr, P. E. Karchin, N. Poudyal, J. Sturdy, P. Thapa, S. Zaleski, M. Brodski, J. Buchanan, C. Caillol, S. Dasu, L. Dodd, S. Duric, B. Gomber, M. Grothe, M. Herndon, A. Hervé, U. Hussain, P. Klabbers, A. Lanaro, A. Levine, K. Long, R. Loveless, T. Ruggles, A. Savin, N. Smith, W. H. Smith, D. Taylor, N. Woods, CMS Collaboration

**Affiliations:** 1 Yerevan Physics Institute, Yerevan, Armenia; 2 Institut für Hochenergiephysik, Wien, Austria; 3 Institute for Nuclear Problems, Minsk, Belarus; 4 Universiteit Antwerpen, Antwerpen, Belgium; 5 Vrije Universiteit Brussel, Brussel, Belgium; 6 Université Libre de Bruxelles, Bruxelles, Belgium; 7 Ghent University, Ghent, Belgium; 8 Université Catholique de Louvain, Louvain-la-Neuve, Belgium; 9 Centro Brasileiro de Pesquisas Fisicas, Rio de Janeiro, Brazil; 10 Universidade do Estado do Rio de Janeiro, Rio de Janeiro, Brazil; 11 Universidade Estadual Paulista, Universidade Federal do ABC, São Paulo, Brazil; 12 Institute for Nuclear Research and Nuclear Energy, Bulgarian Academy of Sciences, Sofia, Bulgaria; 13 University of Sofia, Sofia, Bulgaria; 14 Beihang University, Beijing, China; 15 Institute of High Energy Physics, Beijing, China; 16 State Key Laboratory of Nuclear Physics and Technology, Peking University, Beijing, China; 17 Tsinghua University, Beijing, China; 18 Universidad de Los Andes, Bogota, Colombia; 19 Faculty of Electrical Engineering, Mechanical Engineering and Naval Architecture, University of Split, Split, Croatia; 20 Faculty of Science, University of Split, Split, Croatia; 21 Institute Rudjer Boskovic, Zagreb, Croatia; 22 University of Cyprus, Nicosia, Cyprus; 23 Charles University, Prague, Czech Republic; 24 Universidad San Francisco de Quito, Quito, Ecuador; 25 Academy of Scientific Research and Technology of the Arab Republic of Egypt, Egyptian Network of High Energy Physics, Cairo, Egypt; 26 National Institute of Chemical Physics and Biophysics, Tallinn, Estonia; 27 Department of Physics, University of Helsinki, Helsinki, Finland; 28 Helsinki Institute of Physics, Helsinki, Finland; 29 Lappeenranta University of Technology, Lappeenranta, Finland; 30 IRFU, CEA, Université Paris-Saclay, Gif-sur-Yvette, France; 31 Laboratoire Leprince-Ringuet, Ecole polytechnique, CNRS/IN2P3, Université Paris-Saclay, Palaiseau, France; 32 Université de Strasbourg, CNRS IPHC UMR 7178, 67000 Strasbourg, France; 33 Centre de Calcul de l’Institut National de Physique Nucleaire et de Physique des Particules, CNRS/IN2P3, Villeurbanne, France; 34 Université de Lyon, Université Claude Bernard Lyon 1, CNRS-IN2P3, Institut de Physique Nucléaire de Lyon, Villeurbanne, France; 35 Georgian Technical University, Tbilisi, Georgia; 36 Tbilisi State University, Tbilisi, Georgia; 37 RWTH Aachen University, I. Physikalisches Institut, Aachen, Germany; 38 RWTH Aachen University, III. Physikalisches Institut A, Aachen, Germany; 39 RWTH Aachen University, III. Physikalisches Institut B, Aachen, Germany; 40 Deutsches Elektronen-Synchrotron, Hamburg, Germany; 41 University of Hamburg, Hamburg, Germany; 42 Institut für Experimentelle Kernphysik, Karlsruhe, Germany; 43 Institute of Nuclear and Particle Physics (INPP), NCSR Demokritos, Aghia Paraskevi, Greece; 44 National and Kapodistrian University of Athens, Athens, Greece; 45 National Technical University of Athens, Athens, Greece; 46 University of Ioánnina, Ioannina, Greece; 47 MTA-ELTE Lendület CMS Particle and Nuclear Physics Group, Eötvös Loránd University, Budapest, Hungary; 48 Wigner Research Centre for Physics, Budapest, Hungary; 49 Institute of Nuclear Research ATOMKI, Debrecen, Hungary; 50 Institute of Physics, University of Debrecen, Debrecen, Hungary; 51 Indian Institute of Science (IISc), Bangalore, India; 52 National Institute of Science Education and Research, Bhubaneswar, India; 53 Panjab University, Chandigarh, India; 54 University of Delhi, Delhi, India; 55 Saha Institute of Nuclear Physics, HBNI, Kolkata, India; 56 Indian Institute of Technology Madras, Madras, India; 57 Bhabha Atomic Research Centre, Mumbai, India; 58 Tata Institute of Fundamental Research-A, Mumbai, India; 59 Tata Institute of Fundamental Research-B, Mumbai, India; 60 Indian Institute of Science Education and Research (IISER), Pune, India; 61 Institute for Research in Fundamental Sciences (IPM), Tehran, Iran; 62 University College Dublin, Dublin, Ireland; 63 INFN Sezione di Bari, Università di Bari, Politecnico di Bari, Bari, Italy; 64 INFN Sezione di Bologna, Università di Bologna, Bologna, Italy; 65 INFN Sezione di Catania, Università di Catania, Catania, Italy; 66 INFN Sezione di Firenze, Università di Firenze, Firenze, Italy; 67 INFN Laboratori Nazionali di Frascati, Frascati, Italy; 68 INFN Sezione di Genova, Università di Genova, Genova, Italy; 69 INFN Sezione di Milano-Bicocca, Università di Milano-Bicocca, Milan, Italy; 70 INFN Sezione di Napoli, Università di Napoli ’Federico II’ , Napoli, Italy, Università della Basilicata, Potenza, Italy, Università G. Marconi, Roma, Italy; 71 INFN Sezione di Padova, Università di Padova, Padova, Italy, Università di Trento, Trento, Italy; 72 INFN Sezione di Pavia, Università di Pavia, Pavia, Italy; 73 INFN Sezione di Perugia, Università di Perugia, Perugia, Italy; 74 INFN Sezione di Pisa, Università di Pisa, Scuola Normale Superiore di Pisa, Pisa, Italy; 75 INFN Sezione di Roma, Sapienza Università di Roma, Rome, Italy; 76 INFN Sezione di Torino, Università di Torino, Turino, Italy, Università del Piemonte Orientale, Novara, Italy; 77 INFN Sezione di Trieste, Università di Trieste, Trieste, Italy; 78 Kyungpook National University, Daegu, Korea; 79 Chonbuk National University, Jeonju, Korea; 80 Chonnam National University, Institute for Universe and Elementary Particles, Kwangju, Korea; 81 Hanyang University, Seoul, Korea; 82 Korea University, Seoul, Korea; 83 Seoul National University, Seoul, Korea; 84 University of Seoul, Seoul, Korea; 85 Sungkyunkwan University, Suwon, Korea; 86 Vilnius University, Vilnius, Lithuania; 87 National Centre for Particle Physics, Universiti Malaya, Kuala Lumpur, Malaysia; 88 Centro de Investigacion y de Estudios Avanzados del IPN, Mexico City, Mexico; 89 Universidad Iberoamericana, Mexico City, Mexico; 90 Benemerita Universidad Autonoma de Puebla, Puebla, Mexico; 91 Universidad Autónoma de San Luis Potosí, San Luis Potosí, Mexico; 92 University of Auckland, Auckland, New Zealand; 93 University of Canterbury, Christchurch, New Zealand; 94 National Centre for Physics, Quaid-I-Azam University, Islamabad, Pakistan; 95 National Centre for Nuclear Research, Swierk, Poland; 96 Faculty of Physics, Institute of Experimental Physics, University of Warsaw, Warsaw, Poland; 97 Laboratório de Instrumentação e Física Experimental de Partículas, Lisban, Portugal; 98 Joint Institute for Nuclear Research, Dubna, Russia; 99 Petersburg Nuclear Physics Institute, Gatchina, St. Petersburg, Russia; 100 Institute for Nuclear Research, Moscow, Russia; 101 Institute for Theoretical and Experimental Physics, Moscow, Russia; 102 Moscow Institute of Physics and Technology, Moscow, Russia; 103 National Research Nuclear University ‘Moscow Engineering Physics Institute’ (MEPhI), Moscow, Russia; 104 P.N. Lebedev Physical Institute, Moscow, Russia; 105 Skobeltsyn Institute of Nuclear Physics, Lomonosov Moscow State University, Moscow, Russia; 106 Novosibirsk State University (NSU), Novosibirsk, Russia; 107 State Research Center of Russian Federation, Institute for High Energy Physics, Protvino, Russia; 108 University of Belgrade, Faculty of Physics and Vinca Institute of Nuclear Sciences, Belgrade, Serbia; 109 Centro de Investigaciones Energéticas Medioambientales y Tecnológicas (CIEMAT), Madrid, Spain; 110 Universidad Autónoma de Madrid, Madrid, Spain; 111 Universidad de Oviedo, Oviedo, Spain; 112 Instituto de Física de Cantabria (IFCA), CSIC-Universidad de Cantabria, Santander, Spain; 113 CERN, European Organization for Nuclear Research, Geneva, Switzerland; 114 Paul Scherrer Institut, Villigen, Switzerland; 115 ETH Zurich - Institute for Particle Physics and Astrophysics (IPA), Zurich, Switzerland; 116 Universität Zürich, Zurich, Switzerland; 117 National Central University, Chung-Li, Taiwan; 118 National Taiwan University (NTU), Taipei, Taiwan; 119 Chulalongkorn University, Faculty of Science, Department of Physics, Bangkok, Thailand; 120 Çukurova University Physics Department, Science and Art Faculty, Adana, Turkey; 121 Middle East Technical University, Physics Department, Ankara, Turkey; 122 Bogazici University, Istanbul, Turkey; 123 Istanbul Technical University, Istanbul, Turkey; 124 Institute for Scintillation Materials of National Academy of Science of Ukraine, Kharkov, Ukraine; 125 National Scientific Center, Kharkov Institute of Physics and Technology, Kharkov, Ukraine; 126 University of Bristol, Bristol, UK; 127 Rutherford Appleton Laboratory, Didcot, UK; 128 Imperial College, London, UK; 129 Brunel University, Uxbridge, UK; 130 Baylor University, Waco, USA; 131 Catholic University of America, Washington, DC, USA; 132 The University of Alabama, Tuscaloosa, USA; 133 Boston University, Boston, USA; 134 Brown University, Providence, USA; 135 University of California, Davis, Davis USA; 136 University of California, Los Angeles, USA; 137 University of California, Riverside, Riverside USA; 138 University of California, San Diego, La Jolla USA; 139 Santa Barbara-Department of Physics, University of California, Santa Barbara, USA; 140 California Institute of Technology, Pasadena, USA; 141 Carnegie Mellon University, Pittsburgh, USA; 142 University of Colorado Boulder, Boulder, USA; 143 Cornell University, Ithaca, USA; 144 Fermi National Accelerator Laboratory, Batavia, USA; 145 University of Florida, Gainesville, USA; 146 Florida International University, Miami, USA; 147 Florida State University, Tallahassee, USA; 148 Florida Institute of Technology, Melbourne, USA; 149 University of Illinois at Chicago (UIC), Chicago, USA; 150 The University of Iowa, Iowa City, USA; 151 Johns Hopkins University, Baltimore, USA; 152 The University of Kansas, Lawrence, USA; 153 Kansas State University, Manhattan, USA; 154 Lawrence Livermore National Laboratory, Livermore, USA; 155 University of Maryland, College Park, USA; 156 Massachusetts Institute of Technology, Cambridge, USA; 157 University of Minnesota, Minneapolis, USA; 158 University of Mississippi, Oxford, USA; 159 University of Nebraska-Lincoln, Lincoln, USA; 160 State University of New York at Buffalo, Buffalo, USA; 161 Northeastern University, Boston, USA; 162 Northwestern University, Evanston, USA; 163 University of Notre Dame, Notre Dame, USA; 164 The Ohio State University, Columbus, USA; 165 Princeton University, Princeton, USA; 166 University of Puerto Rico, Mayaguez, USA; 167 Purdue University, West Lafayette, USA; 168 Purdue University Northwest, Hammond, USA; 169 Rice University, Houston, USA; 170 University of Rochester, Rochester, USA; 171 The Rockefeller University, New York, USA; 172 Rutgers, The State University of New Jersey, Piscataway, USA; 173 University of Tennessee, Knoxville, USA; 174 Texas A&M University, College Station, USA; 175 Texas Tech University, Lubbock, USA; 176 Vanderbilt University, Nashville, USA; 177 University of Virginia, Charlottesville, USA; 178 Wayne State University, Detroit, USA; 179 University of Wisconsin-Madison, Madison, WI USA; 180 CERN, 1211 Geneva 23, Switzerland

## Abstract

A search for new physics in events with a Z boson produced in association with large missing transverse momentum at the LHC is presented. The search is based on the 2016 data sample of proton-proton collisions recorded with the CMS experiment at $\sqrt{s} = 13\,\text {TeV}$, corresponding to an integrated luminosity of 35.9$\,\text {fb}^{-1}$. The results of this search are interpreted in terms of a simplified model of dark matter production via spin-0 or spin-1 mediators, a scenario with a standard-model-like Higgs boson produced in association with the Z boson and decaying invisibly, a model of unparticle production, and a model with large extra spatial dimensions. No significant deviations from the background expectations are found, and limits are set on relevant model parameters, significantly extending the results previously achieved in this channel.

**Electronic supplementary material:**

The online version of this article (10.1140/epjc/s10052-018-5740-1) contains supplementary material, which is available to authorized users.

## Introduction

In the pursuit of new physics at the CERN LHC, many scenarios have been proposed in which production of particles that leave no trace in collider detectors is accompanied also by production of a standard model (SM) particle, which balances the transverse momentum in an event. The final state considered in this analysis is the production of a pair of leptons ($\ell ^{+}\ell ^{-}$, where $\ell =\mathrm {e}$ or $\mathrm {\mu }$), consistent with originating from a $\text{ Z }$ boson, together with large missing transverse momentum ($p_{\mathrm {T}} ^\text {miss}$). This final state is well-suited to probe such beyond the SM (BSM) scenarios, as it has relatively small and precisely known SM backgrounds.

One of the most significant puzzles in modern physics is the nature of dark matter (DM). In the culmination of over a century of observations, the “${\varLambda }_{\mathrm {CDM}}$” standard model of cosmology has established that, in the total cosmic energy budget, known matter only accounts for about 5%, DM corresponds to 27%, and the rest is dark energy [1]. Although several astrophysical observations indicate that DM exists and interacts gravitationally with known matter, there is no evidence yet for nongravitational interactions between DM and SM particles. While the nature of DM remains a mystery, there are a number of models that predict a particle physics origin. If DM particles exist, they can possibly be produced directly from, annihilate into, or scatter off SM particles. Recent DM searches have exploited various methods including direct [2] and indirect [3] detection. If DM can be observed in direct detection experiments, it must have substantial couplings to quarks and/or gluons, and could also be produced at the LHC [4–9].

A promising possibility is that DM may take the form of weakly interacting massive particles. The study presented here considers one possible mechanism for producing such particles at the LHC [10]. In this scenario, a $\text{ Z }$ boson, produced in proton-proton (pp) collisions, recoils against a pair of DM particles, $\chi \overline{\chi }$. The $\text{ Z }$ boson subsequently decays into two charged leptons, producing a low-background dilepton signature, together with $p_{\mathrm {T}} ^\text {miss}$ due to the undetected DM particles. In this analysis, the DM particle $\chi$ is assumed to be a Dirac fermion. Four simplified models of DM production via an *s*-channel mediator exchange are considered. In these models, the mediator has a spin of 1 (0) and vector or axial-vector (scalar or pseudoscalar) couplings to quarks and DM particles. The free parameters of each model are the masses $m_\text {med}$ and $m_\mathrm {DM}$ of the mediator and DM particle, respectively, as well as the coupling constant $g_{\mathrm {q}}$ ($g_\mathrm {DM}$) between the mediator and the quarks (DM particles). The vector coupling model can be described with the following Lagrangian:

$$\begin{aligned} \mathcal {L}_{\text {vector}} = g_\mathrm {DM} {Z'}_{\mu }\overline{\chi }\gamma ^{\mu }\chi + g_{\mathrm {q}} \sum _{\mathrm {q}} {Z'}_{\mu } \overline{\mathrm {q}}\gamma ^{\mu }\mathrm {q}, \end{aligned}$$

where the spin-1 mediator is denoted as $\text{ Z } '$ and the SM quark fields are referred to as $\text{ q }$ and $\overline{\text{ q }}$. The Lagrangian for an axial-vector coupling is obtained by making the replacement $\gamma ^\mu \rightarrow \gamma ^5\gamma ^\mu$. In the case of a spin-0 mediator $\phi$, the couplings between mediator and quarks are assumed to be Yukawa-like, with $g_{\mathrm {q}}$ acting as a multiplicative modifier for the SM Yukawa coupling ${y_{\mathrm {q}} = \sqrt{2}m_{\mathrm {q}}/v}$ (where $v = 246 \,\text {GeV}$ is the SM Higgs field vacuum expectation value), leading to the Lagrangian:

$$\begin{aligned} \mathcal {L}_{\text {scalar}} = g_\mathrm {DM} {\phi }\overline{\chi }\chi + g_{\mathrm {q}} \frac{\phi }{\sqrt{2}}\sum _{\mathrm {q}} y_{\mathrm {q}} \overline{\mathrm {q}}\mathrm {q}. \end{aligned}$$

**Fig. 1:**
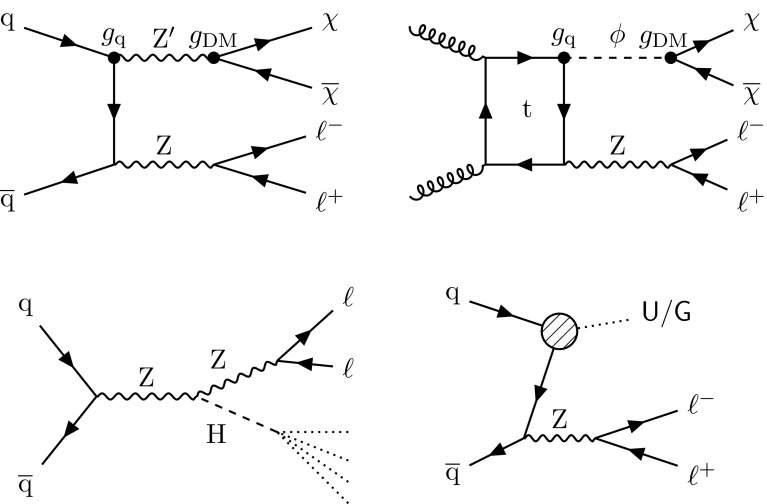
Feynman diagrams illustrative of the processes beyond the SM considered in this paper: (upper left) DM production in a simplified model with a spin-1 mediator $\text{ Z } '$; (upper right) DM production in a simplified model with a spin-0 mediator $\phi$; (lower left) production of a Higgs boson in association with Z boson with subsequent decay of the Higgs boson into invisible particles; (lower right) unparticle or graviton production. The diagrams were drawn using the TikZ-Feynman package [11]

The Lagrangian with pseudoscalar couplings is obtained by inserting a factor of $i\gamma ^5$ into each of the two terms (i.e., ${\bar{\chi }}\chi \rightarrow i{\bar{\chi }}\gamma ^5\chi$ and ${\bar{\mathrm {q}}} \mathrm {q}\rightarrow i{\bar{\mathrm {q}}}\gamma ^5 \mathrm {q}$). Example diagrams of DM production via spin-1 and spin-0 mediators are shown in Fig. 1 (upper left and right, respectively).

A primary focus of the LHC physics program after the discovery of a Higgs boson (H) [12–14] by the ATLAS and CMS Collaborations is the study of the properties of this new particle. The observation of a sizable branching fraction of the Higgs boson to invisible states [15–17] would be a strong sign of BSM physics. Supersymmetric (SUSY) models embodying R-parity conservation contain a stable neutral lightest SUSY particle (LSP), e.g., the lightest neutralino [18], leading to the possibility of decays of the Higgs boson into pairs of LSPs. Certain models with extra spatial dimensions predict graviscalars that could mix with the Higgs boson [19]. As a consequence, the Higgs boson could oscillate to a graviscalar and disappear from the SM brane. The signature would be equivalent to an invisible decay of the Higgs boson. There could also be contributions from Higgs boson decays into graviscalars [20]. With the same effect as the simplified DM models presented earlier, “Higgs portal” models [21–23] construct a generic connection between SM and DM particles via a Higgs boson mediator. This analysis considers decays into invisible particles of an SM-like Higgs boson produced in association with a $\text{ Z }$ boson, as shown in Fig. 1 (lower left).

Another popular BSM paradigm considered here is the Arkani-Hamed–Dimopoulos–Dvali (ADD) model with large extra spatial dimensions [24–26], which is motivated by the hierarchy problem, i.e., the disparity between the electroweak unification scale ($M_\mathrm {EW} \sim 1\,\text {TeV}$) and the Planck scale ($M_\mathrm {Pl} \sim 10^{16}\,\text {TeV}$). This model predicts graviton ($\text{ G }$) production via the process $\text{ q } \overline{\text{ q }} \rightarrow \text{ Z } + \text{ G }$. The graviton escapes detection, leading to a mono-$\text{ Z }$ signature (Fig. 1, lower right). In the ADD model, the apparent Planck scale in four space-time dimensions is given by $M_\mathrm {Pl}^2 \approx M_\mathrm {D}^{n+2}R^n$, where $M_\mathrm {D}$ is the true Planck scale of the full *n*+4 dimensional space-time and *R* is the compactification radius of the extra dimensions. Assuming $M_\mathrm {D}$ is of the same order as $M_\mathrm {EW}$, the observed large value of $M_\mathrm {Pl}$ points to an *R* of order 1 mm to 1 fm for 2 to 7 extra dimensions. The consequence of the large compactification scale is that the mass spectrum of the Kaluza–Klein graviton states becomes nearly continuous, resulting in a broad $\text{ Z }$ boson transverse momentum ($p_{\mathrm {T}}$) spectrum.

The final BSM model considered in this analysis is the phenomenologically interesting concept of unparticles, which appear in the low-energy limit of conformal field theories. In the high-energy regime, a new, scale invariant Banks–Zaks field with a nontrivial infrared fixed point is introduced [27]. The interaction between the SM and Banks–Zaks sectors is mediated by particles of large mass scale $M_{\textsf {U}}$, below which the interaction is suppressed and can be treated via an effective field theory (EFT). The low-energy regime will include unparticles, which have phase space factors equivalent to those of a noninteger number of ordinary particles [28–30]. In this analysis, the emission of spin-0 unparticles from SM quarks is considered. Because of the weakness of the unparticle interactions with the SM fields, the unparticle evades detection. The EFT Lagrangian used to interpret the results is defined as follows:

$$\begin{aligned} \mathcal {L}_{U} = \frac{\lambda }{{\varLambda }_{\textsf {U}} ^{d_{\textsf {U}}-1}} \mathcal {O}_{\textsf {U}} \overline{\text{ q }}\text{ q }, \end{aligned}$$

where $\lambda$ represents the coupling between the SM and unparticle fields, ${\varLambda }_{\textsf {U}}$ is the cutoff scale of the EFT, and $d_{\textsf {U}}$ is the characteristic scaling dimension of the theory. The unparticle operator is denoted as $\mathcal {O}_{\textsf {U}}$. A representative Feynman diagram of the interaction is shown in Fig. 1 (lower right).

The search described in this paper is based on a data set recorded with the CMS detector in 2016, which corresponds to an integrated luminosity of $35.9 \pm 0.9{\,\text {fb}^{-1}}$ of pp collisions at a center-of-mass energy of 13$\,\text {TeV}$.

The paper is organized as follows: after a brief review of previous work in Sect. 2, followed by a description of the CMS detector in Sect. 3, we discuss the background composition in Sect. 4. Simulated samples are reviewed in Sect. 5, followed by the event reconstruction and event selection description in Sects. 6 and 7, respectively. The details of the background estimation are given in Sect. 8. The multivariate analysis of invisible Higgs boson decays is summarized in Sect. 9, followed by the discussion of selection efficiencies and systematic uncertainties in Sect. 10. The results are given in Sect. 11, and Sect. 12 summarizes the paper.

## Review of previous work

A search by the CMS Collaboration in the same topology using an earlier data set corresponding to an integrated luminosity of 2.3$\,\text {fb}^{-1}$ of pp collisions collected in 2015 at $\sqrt{s}=13\,\text {TeV}$ found no evidence for BSM physics [31]. In addition to the order of magnitude increase in the integrated luminosity, significant differences with respect to the previous analysis include: new techniques for estimating irreducible backgrounds, which were not viable with the previous data set; improvements in the event selection; and a broader range of BSM models probed.

In the previous CMS result [31], under the same simplified model assumptions as used in this paper, DM mediator masses of up to 290 (300)$\,\text {GeV}$ were excluded for fixed vector (axial-vector) couplings of $g_{\mathrm {q}}=0.25$ and $g_\mathrm {DM}=1.0$. Here and in what follows all limits are given at 95% confidence level (CL), unless explicitly specified otherwise. Similar DM models have been also probed in the $\gamma$+$p_{\mathrm {T}} ^\text {miss}$  [32] and jet+$p_{\mathrm {T}} ^\text {miss}$  [33] topologies at $\sqrt{s} = 13\,\text {TeV}$ by the ATLAS Collaboration, excluding mediators with vector (axial-vector) couplings up to masses of 1.2 (1.25) $\,\text {TeV}$. The most stringent limits on DM production in this context were obtained in a CMS analysis of events with a jets+$p_{\mathrm {T}} ^\text {miss}$ topology performed on a subset of the present data set, corresponding to an integrated luminosity of 12.9$\,\text {fb}^{-1}$  [34]. In that analysis, mediator masses of up to 1.95$\,\text {TeV}$ were excluded for both vector and axial-vector couplings. In the case of a scalar mediator coupled only to quarks and DM particles with $g_{\mathrm {q}} = g_\mathrm {DM} = 1$, no exclusion was set. For the pseudoscalar mediator, under the same assumptions, masses below 430$\,\text {GeV}$ were excluded.

Invisible decays of the SM Higgs boson—hereafter H(inv.)—have been targeted by both ATLAS and CMS. These searches used both the Z+$p_{\mathrm {T}} ^\text {miss}$ and jets+$p_{\mathrm {T}} ^\text {miss}$ topologies, the latter including gluon fusion and vector fusion processes as well as associated production with a vector boson reconstructed as a single jet. The most stringent constraints were obtained from a combination of searches in these final states at $\sqrt{s} = 8\,\text {TeV}$ by ATLAS [35] and at multiple center-of-mass energies by CMS [36], which, under the assumption of SM production, exclude a branching fraction for H(inv.) decays larger than $25\%$ for ATLAS and $24\%$ for CMS.

Real emission of gravitons in the ADD scenario has been most recently probed in the jet+$p_{\mathrm {T}} ^\text {miss}$ topology by CMS at $\sqrt{s} = 8\,\text {TeV}$ [37] and by ATLAS at $\sqrt{s} = 13\,\text {TeV}$ [38]. In these analyses, the fundamental Planck scale $M_\mathrm {D}$ of the *n*+4 dimensional theory has been constrained to be larger than 3.3–5.6$\,\text {TeV}$ (CMS) and 4.1–6.6$\,\text {TeV}$ (ATLAS), for the number of extra dimensions between 6 and 2. Previous CMS analyses in the same final state as this analysis have excluded unparticle cutoff scales from $400\,\text {GeV}$ at large values of the scaling dimension $d_{\textsf {U}} =2.2$, up to hundreds of $\,\text {TeV}$ at low values of $d_{\textsf {U}} \approx 1$ [31, 39].

## The CMS detector

The central feature of the CMS apparatus is a superconducting solenoid of 6$\text {\,m}$ internal diameter, providing a magnetic field of 3.8$\text {\,T}$. Within the solenoid volume are a silicon pixel and strip tracker, a lead tungstate crystal electromagnetic calorimeter (ECAL), and a brass and scintillator hadron calorimeter (HCAL), each composed of a barrel and two endcap sections. Forward calorimeters extend the pseudorapidity coverage provided by the barrel and endcap detectors. Muons are detected in gas-ionization chambers embedded in the steel flux-return yoke outside the solenoid.

Events of interest are selected using a two-tiered trigger system [40]. The first level, composed of custom hardware processors, uses information from the calorimeters and muon detectors to select events at a rate of around 100$\text {\,kHz}$ within a time interval of less than 4$\,\mu \text {s}$. The second level, known as the high-level trigger, consists of a farm of processors running a version of the full event reconstruction software optimized for fast processing, and reduces the event rate to around 1$\text {\,kHz}$ before data storage.

A more detailed description of the CMS detector, together with a definition of the coordinate system used and the relevant kinematic variables, can be found in Ref. [41].

## Background composition

Several SM processes can produce the dilepton+$p_{\mathrm {T}} ^\text {miss}$ final state. Since none of the BSM physics signals probed in this analysis are expected to produce a resonance peak in the $p_{\mathrm {T}} ^\text {miss}$ distribution, adequate modeling of each SM background process is necessary. The following SM background processes have been considered in this analysis:

- $\text{ Z } \text{ Z } \rightarrow 2\ell 2\nu$ production, which yields the same final state as the signal and contributes approximately 60% of the total background.
- $\mathrm {W}\text{ Z } \rightarrow \ell \nu \ell \ell$ production, where the lepton from the $\mathrm {W}$ boson decay is not identified either because it fails the lepton identification, or because it falls outside the detector acceptance or kinematic selections. This process contributes approximately 25% of the total background, and the kinematic distributions are similar to those for the $\text{ Z } \text{ Z } \rightarrow 2\ell 2\nu$ process.
- $\mathrm {W}\mathrm {W}\rightarrow \ell \nu \ell \nu$ events, where the dilepton invariant mass falls into the $\text{ Z }$ boson mass window. These events constitute approximately 5% of the background.
- Events with leptonically decaying top quarks (mostly ${\text{ t }\overline{\text{ t }}}$ and $\text{ t } \mathrm {W}$), where the dilepton invariant mass falls into the $\text{ Z }$ boson mass window, and which contribute about 5% of the total background.
- Drell–Yan (DY) production, $\text{ Z }/\gamma ^*\rightarrow \ell \ell$, which can produce events with large $p_{\mathrm {T}} ^\text {miss}$ caused mainly by jet energy mismeasurement and detector acceptance effects. It amounts to approximately 5% of the total background.
- Triboson processes (e.g., $\mathrm {W}\mathrm {W}\mathrm {W}$), which have a small cross section and contribute less than 1% of the total background.

Processes that were found to have a negligible contribution to the signal region include: $\mathrm {W}$+jets, because of the very low probability for a jet to be reconstructed as a lepton and the dilepton system to be within the $\text{ Z }$ boson mass window; the SM process $\text{ Z } (\rightarrow \ell \ell )\text{ H } (\rightarrow \text{ Z } \text{ Z } \rightarrow 4\nu )$, which is a subset of the $\text{ Z } \text{ H } (\text {inv.})$ signal and accounts for 0.1% of SM Higgs boson decays; and ${\mathrm{gg} \rightarrow \text{ H } (\rightarrow \mathrm {W}\mathrm {W})}$, which has similar topology to continuum $\mathrm {W}\mathrm {W}$ production but makes a negligible contribution after the full selection.

## Simulation

Simulated Monte Carlo (MC) samples are used to estimate backgrounds, to validate the background estimation techniques using control samples in data, to calculate signal efficiency, and to optimize the analysis.

Diboson production (VV, where $\mathrm {V}=$ W or Z) via $\text{ q } \overline{\text{ q }}$ annihilation, as well as $\text{ Z } \text{ H }$ production via $\text{ q } \overline{\text{ q }}$ annihilation and gluon fusion, are generated at next-to-leading order (NLO) in quantum chromodynamics (QCD) with powheg 2.0 [42–45]. The $\mathrm{gg \rightarrow \mathrm {W}\mathrm {W}}$ and $\mathrm{gg \rightarrow \text{ Z } \text{ Z }}$ processes are simulated at NLO with mcfm v7.01 [46]. The $\text{ Z }$+jets, $\text{ Z } \gamma$, ${\text{ t }\overline{\text{ t }}}$, ${\text{ t }\overline{\text{ t }}} \mathrm {V}$, and $\mathrm {VVV}$ samples are generated at NLO with either MadGraph 5_amc@nlo v2.3.2 [47] or powheg.

Samples of DM particle production in the simplified model framework are generated using DmSimp [48–50] interfaced with MadGraph 5_amc@nlo v2.4.3. Samples are generated over a range of values for the masses $m_\text {med}$ and $m_\mathrm {DM}$. For the vector and axial-vector models, samples are generated at NLO in QCD with up to one additional parton in the matrix element calculations, and the mediator couplings to the SM and DM fields are set to $g_{\mathrm {q}} = 0.25$ and $g_\mathrm {DM} = 1$, respectively. For the scalar and pseudoscalar models, samples are generated at leading order in QCD, and the couplings are set to $g_{\mathrm {q}} = g_\mathrm {DM} = 1$. This choice of couplings is recommended by the ATLAS/CMS dark matter forum [10] and by the LHC dark matter working group [51]. For all DM particle production samples, the central values of the renormalization and factorization scales are set to the $m_\mathrm {T}^{2}$ scale after $k_\mathrm {T}$-clustering of the event.

Events for the ADD scenario of large extra dimensions and for the unparticle model are generated at leading order (LO) using an EFT implementation in pythia 8.205  [52–54]. In the ADD case, event samples are produced for $M_\mathrm {D} = 1$, 2, and 3 $\,\text {TeV}$, each with $n =$ 2–7. In order to ensure the validity of the EFT, the signal is truncated for $\hat{s} > M_\mathrm {D}^2$, where $\hat{s}$ is the center-of-mass energy squared of the incoming partons. Events above this threshold are suppressed by an additional weight of $M_\mathrm {D}^4 / \hat{s}^2$. In general, this procedure has a larger effect for large values of *n*, for which the distribution of $\hat{s}$ is shifted towards higher values [53]. For the unparticle case, samples are generated for scaling dimensions $d_{\textsf {U}}$ between 1.01 and 2.2, with the cutoff scale ${\varLambda }_{\textsf {U}}$ set to $15 \,\text {TeV}$ and the coupling $\lambda$ set to 1. Since both ${\varLambda }_{\textsf {U}}$ and $\lambda$ modify the cross sections of the signal prediction, but not its kinematic distributions [54], a simple rescaling of cross sections is performed to obtain signal predictions for alternative values of these parameters. No truncation is performed for the unparticle signal so that the results can be compared with those of previous searches.

In all cases, pythia versions 8.205 or higher is used for parton showering, hadronization, and the underlying event simulation, using tune CUETP8M1 [55]. The merging of jets from matrix element calculations and parton shower descriptions is done using the MLM [56] (FxFx [57]) scheme for LO (NLO) samples. The NNPDF3.0 [58] parton distribution function (PDF) set is used, with the order corresponding to the one used for the signal or background simulation.

For all MC samples, the detector response is simulated using a detailed description of the CMS detector, based on the ${\textsc {Geant4}}$ package [59]. Minimum bias events are superimposed on the simulated events to emulate the additional pp interactions per bunch crossing (pileup). All MC samples are corrected to reproduce the pileup distribution as measured in the data. The average number of pileup events per bunch crossing is approximately 23 in the data sample analyzed.

## Event reconstruction

In this analysis, the particle-flow (PF) event reconstruction algorithm [60] is used. The PF algorithm is designed to leverage information from all CMS detector components to reconstruct and identify individual particles, namely: electrons, muons, photons, and charged and neutral hadrons. The reconstructed vertex with the largest value of summed physics-object $p_{\mathrm {T}} ^2$ is taken to be the primary $\mathrm {p}\mathrm {p}$ interaction vertex. The physics objects are the track-jets, clustered using the jet finding algorithm [61, 62] with the tracks assigned to the vertex as inputs, and the associated missing transverse momentum, taken as the negative vector sum of the $p_{\mathrm {T}}$ of those jets.

Electron candidates are reconstructed using an algorithm that combines information from the ECAL, HCAL, and the tracker [63]. To reduce the electron misidentification rate, electron candidates are subjected to additional identification criteria, which are based on the distribution of the electromagnetic shower in the ECAL, the relative amount of energy deposited in the HCAL in the cluster, a matching of the trajectory of an electron track with the cluster in the ECAL, and its consistency with originating from the selected primary vertex. Candidates that are identified as originating from photon conversions in the detector material are removed.

Muon candidate reconstruction is based on two main algorithms: in the first, tracks in the silicon tracker are matched to track stubs (or segments) reconstructed in the muon detectors; in the second algorithm, a combined fit is performed to signals in both the silicon tracker and the muon system [64]. The two resulting collections are merged, with the momentum measurement of the latter algorithm taking precedence. To reduce the muon misidentification rate, further identification criteria are applied on the basis of the number of measurements in the tracker and in the muon system, the quality of the muon track fit, and its consistency with the selected primary vertex location.

Leptons produced in the decay of $\text{ Z }$ bosons are expected to be isolated from hadronic activity in the event. The isolation is defined from the sum of the momenta of all PF candidates found in a cone of radius $R=\sqrt{\smash [b]{({\varDelta }\eta )^2+({\varDelta }\phi )^2}} = 0.4$ built around each lepton, where ${\varDelta }\phi$ and ${\varDelta }\eta$ are, respectively, the differences in the azimuthal angle (measured in radians) and in the pseudorapidity between the lepton and the PF candidate. The contribution to the isolation from the lepton candidate itself is removed. For muons, the isolation sum is required to be smaller than 15% of the muon $p_{\mathrm {T}}$. For electrons in the ECAL barrel (endcap), the limit on this isolation sum is 6.9 (8.2)% of the electron $p_{\mathrm {T}}$. In order to mitigate the dependence of the isolation variable on the number of pileup interactions, charged hadrons are included in the sum only if they are consistent with originating from the selected primary vertex of the event. To correct for the contribution to the isolation sum of neutral hadrons and photons from pileup interactions, different strategies are adopted for electrons and muons. For electrons, a median energy density ($\rho$) is determined on an event-by-event basis using the method described in Ref. [65]. The contribution of the pileup particles is then estimated as a product of $\rho$ and the effective area of the isolation cone and is subtracted from the isolation sum. For muon candidates, the correction is performed instead by subtracting half the sum of the $p_{\mathrm {T}}$ of the charged-hadron candidates in the cone of interest, which are not associated with the primary vertex. The factor of one half corresponds to the average ratio of neutral to charged particles in pileup interactions.

Jets are constructed from PF candidates using the anti-$k_{\mathrm {T}}$ clustering algorithm [61] with a distance parameter $R = 0.4$, as implemented in the fastjet package [62, 66]. The jet momentum is defined as the vectorial sum of all PF candidate momenta assigned to the jet, and is found in the simulation to be within 5 to 10% of the true momentum over the entire $p_{\mathrm {T}}$ range and detector acceptance used in this analysis. An overall energy subtraction is applied to correct for the extra energy clustered in jets due to pileup interactions, following the procedure in Refs. [65, 67]. Corrections to the jet energy scale and resolution are derived from measurements both in simulation and in data of the energy balance in dijet, multijet, $\gamma$+jet, and leptonic Z+jet events [68, 69].

The missing transverse momentum vector, ${\vec p}_{\mathrm {T}}^{\text {miss}}$, is defined as the projection of the negative vector sum of the momenta of all reconstructed PF candidates in an event onto the plane perpendicular to the beams. Its magnitude is referred to as $p_{\mathrm {T}} ^\text {miss}$. Several event-level filters are applied to discard events with anomalous $p_{\mathrm {T}} ^\text {miss}$ arising from specific well-understood issues with the detector components or event reconstruction [70]. Jet energy corrections are propagated to the missing transverse momentum by adjusting the momentum of the PF candidate constituents of each reconstructed jet.

For the purpose of rejecting events involving top quark production, jets originating from b quark fragmentation (b jets) are identified by “b tagging.” The b tagging technique employed is based on the “combined secondary vertex” CSVv2 algorithm [71, 72]. The algorithm is calibrated to provide, on average, 80% efficiency for tagging jets originating from b quarks, and 10% probability of light-flavor jet misidentification.

For the purpose of rejecting events containing $\tau$ leptons, hadronically decaying $\tau$ leptons ($\tau _\mathrm {h}$) are identified using the “hadron-plus-strips” algorithm [73]. The algorithm identifies a jet as a $\tau _\mathrm {h}$ candidate if a subset of the particles assigned to the jet is consistent with the hadronic decay products of a $\tau$ lepton [73]. In addition, $\tau _\mathrm {h}$ candidates are required to be isolated from other activity in the event.

## Event selection

Events with electrons (muons) are collected using dielectron (dimuon) triggers, with the thresholds of $p_{\mathrm {T}} > 23$ (17)$\,\text {GeV}$ and $p_{\mathrm {T}} > 12$ (8)$\,\text {GeV}$ for the leading and subleading electron (muon), respectively. Single-electron and single-muon triggers (with $p_{\mathrm {T}}$ thresholds of 27 and 24$\,\text {GeV}$, respectively) are also used in order to recover residual trigger inefficiencies.

Events are required to have exactly two ($N_{\ell } = 2$) well-identified, isolated leptons of the same flavor and opposite electric charge ($\mathrm {e}^+\mathrm {e}^-$ or $\mathrm {\mu ^+}\mathrm {\mu ^-}$). The leading electron (muon) of the pair must have $p_{\mathrm {T}} > 25$ (20)$\,\text {GeV}$, while $p_{\mathrm {T}} > 20\,\text {GeV}$ is required for the subleading lepton. The dilepton invariant mass is required to be within 15$\,\text {GeV}$ of the established $\text{ Z }$ boson mass $m_\mathrm{Z}$ [74]. The dilepton $p_{\mathrm {T}}$ ($p_{\mathrm {T}} ^{\, \ell \ell }$) must be larger than 60$\,\text {GeV}$ to reject the bulk of the $\text{ Z }/\gamma ^*\rightarrow \ell \ell$ background. Since little hadronic activity is expected in this final state, events having more than one jet with $p_{\mathrm {T}} >30~\,\text {GeV}$ are rejected. The top quark background is suppressed by applying a b jet veto: events with at least one b-tagged jet with $p_{\mathrm {T}} > 20\,\text {GeV}$ reconstructed within the tracker acceptance, $|\eta | < 2.4$, are removed. To reduce the $\mathrm {W}\text{ Z }$ background in which both bosons decay leptonically, events containing additional electrons (muons) with $p_{\mathrm {T}} > 10$ (5)$\,\text {GeV}$ and events with loosely identified hadronically decaying $\tau$ leptons ($\tau _\mathrm {h}$) with $p_\mathrm {T}>18\,\text {GeV}$ are removed.

The event selection is optimized using three variables: the $p_{\mathrm {T}} ^\text {miss}$, the azimuthal angle formed between the dilepton $p_{\mathrm {T}}$ and the missing transverse momentum vector, ${\varDelta }\phi ({\vec p}_{\mathrm {T}} ^{\, \ell \ell },{\vec p}_{\mathrm {T}}^{\text {miss}})$, and the $p_{\mathrm {T}} ^\text {miss}$-$p_{\mathrm {T}} ^{\, \ell \ell }$ balance ratio, $|p_{\mathrm {T}} ^\text {miss}-p_{\mathrm {T}} ^{\, \ell \ell }|/p_{\mathrm {T}} ^{\, \ell \ell }$. The latter two variables are powerful in suppressing reducible background processes, such as DY and top quark production. The selection criteria applied to these variables are optimized in order to obtain the best expected signal sensitivity for a wide range of DM parameters that are considered. For each possible set of selections, the full analysis is repeated, including the estimation of backgrounds from control samples in data and the systematic uncertainties. The final selection criteria obtained after optimization are: $p_{\mathrm {T}} ^\text {miss} > 100\,\text {GeV}$, ${\varDelta }\phi ({\vec p}_{\mathrm {T}} ^{\, \ell \ell },{\vec p}_{\mathrm {T}}^{\text {miss}}) > 2.6$ rad, and $|p_{\mathrm {T}} ^\text {miss}-p_{\mathrm {T}} ^{\, \ell \ell }|/p_{\mathrm {T}} ^{\, \ell \ell } < 0.4$.

To avoid positive biases in the $p_{\mathrm {T}} ^\text {miss}$ calculation due to jet mismeasurement, in events with one jet a threshold is applied on the azimuthal angle between this jet and the missing transverse momentum, ${\varDelta }\phi ({\vec p}_{\mathrm {T}} ^{\, j},{\vec p}_{\mathrm {T}}^{\text {miss}}) > 0.5\text {\,rad}$. To reduce the contribution from backgrounds such as $\mathrm {W}\mathrm {W}$ and ${\text{ t }\overline{\text{ t }}}$, a requirement on the distance between the two leptons in the $(\eta ,\phi )$ plane, ${\varDelta }R_{\ell \ell } < 1.8$, is applied.

There are two types of analyses performed in this paper. The main analysis method is based on fitting the $p_{\mathrm {T}} ^\text {miss}$ spectrum in data after applying the above selection criteria defining the signal region (SR). For the specific interpretation of this analysis involving invisible decays of the SM (125$\,\text {GeV}$) Higgs boson, a multivariate boosted decision tree (BDT) classifier is employed to increase the sensitivity of the analysis. We use the following set of twelve variables to train a multiclass BDT classifier:

- $$\left| m_{\ell \ell }-m_{\text{ Z }}\right| $$ (dilepton mass);
- $p_{\mathrm {T}} ^{\ell 1}$ (leading lepton transverse momentum);
- $p_{\mathrm {T}} ^{\ell 2}$ (subleading lepton transverse momentum);
- $p_{\mathrm {T}} ^{\, \ell \ell }$ (dilepton transverse momentum);
- $| \eta ^{\ell 1} |$ (leading lepton pseudorapidity);
- $| \eta ^{\ell 2} |$ (subleading lepton pseudorapidity);
- $p_{\mathrm {T}} ^\text {miss}$ (missing transverse momentum);
- $m_{T}(p_{\mathrm {T}} ^{\ell 1},$ $p_{\mathrm {T}} ^\text {miss}$) (leading lepton transverse mass);
- $m_{T}(p_{\mathrm {T}} ^{\ell 2},$ $p_{\mathrm {T}} ^\text {miss}$) (subleading lepton transverse mass);
- ${\varDelta }\phi ({\vec p}_{\mathrm {T}} ^{\, \ell \ell },{\vec p}_{\mathrm {T}}^{\text {miss}})$ (azimuthal separation between dilepton and missing momentum);
- ${\varDelta }R_{\ell \ell }$ (separation between leptons); and
- $| \cos \theta ^\mathrm{CS}_{\ell 1} |$ (cosine of the polar angle in the Collins–Soper frame [75] for the leading lepton).

Several classes of event samples are considered for the multiclass BDT: ZH(inv.) signal; ZZ; WZ; DY; and flavor-symmetric or nonresonant backgrounds. A BDT is trained targeting each class, and the final discriminator is taken to be the likelihood assigned to ZH(inv.) production, normalized to the sum of the likelihoods of all processes. The SR selection for the BDT analysis is slightly altered from that of the $p_{\mathrm {T}} ^\text {miss}$-based analysis: the dilepton mass requirement is relaxed to be within $30\,\text {GeV}$ of the $\text{ Z }$ boson mass, and the selections on ${\varDelta }\phi ({\vec p}_{\mathrm {T}} ^{\, \ell \ell },{\vec p}_{\mathrm {T}}^{\text {miss}})$, $|p_{\mathrm {T}} ^\text {miss}-p_{\mathrm {T}} ^{\, \ell \ell }|/p_{\mathrm {T}} ^{\, \ell \ell }$, and ${\varDelta }R_{\ell \ell }$ are omitted. The selection for training the BDT additionally requires the missing transverse momentum to be greater than $130\,\text {GeV}$, where differentiating between the diboson background and signal is most challenging. The BDT performance in the untrained region of $100\le p_{\mathrm {T}} ^\text {miss} \le 130\,\text {GeV}$ is found to be adequate, whereas a BDT trained on event samples including this region was found to have significantly degraded performance in the $p_{\mathrm {T}} ^\text {miss} >130\,\text {GeV}$ region.

A summary of the selection criteria for the SR of both the $p_{\mathrm {T}} ^\text {miss}$-based analysis and the BDT analysis is given in Table 1.

**Table 1:** Summary of the kinematic selections for the signal region of both the the $p_{\mathrm {T}} ^\text {miss}$-based analysis and the BDT analysis. Where the selections for the two analyses differ, the BDT requirement is given in parentheses

Selection	Requirement	Reject
$N_{\ell }$	${=}2$	$\mathrm {W}\text{ Z }$, $\mathrm {V}\mathrm {V}\mathrm {V}$
$p_{\mathrm {T}} ^{\ell }$	${>}25/20\,\text {GeV}$ for electrons	QCD
	${>}20\,\text {GeV}$ for muons	
$\text{ Z }$ boson mass requirement	$\left| m_{\ell \ell }-m_{\text{ Z }}\right| < 15\ (30)\,\text {GeV}$	$\mathrm {W}\mathrm {W}$, top quark
Jet counting	${\le }1$ jet with $p_{\mathrm {T}} ^{\, j} > 30\,\text {GeV}$	$\text{ Z }/\gamma ^*\rightarrow \ell \ell$, top quark, $\mathrm {V}\mathrm {V}\mathrm {V}$
$p_{\mathrm {T}} ^{\, \ell \ell }$	${>}60\,\text {GeV}$	$\text{ Z }/\gamma ^*\rightarrow \ell \ell$
b tagging veto	CSVv2 < 0.8484	Top quark, $\mathrm {V}\mathrm {V}\mathrm {V}$
$\tau$ lepton veto	0 $\tau _\mathrm{h}$ cand. with $p_{\mathrm {T}} ^{\tau }>18\,\text {GeV}$	$\mathrm {W}\text{ Z }$
$p_{\mathrm {T}} ^\text {miss}$	${>}100\,\text {GeV}$ ($130\,\text {GeV}$, training only)	$\text{ Z }/\gamma ^*\rightarrow \ell \ell$, $\mathrm {W}\mathrm {W}$, top quark
${\varDelta }\phi ({\vec p}_{\mathrm {T}} ^{\, j},{\vec p}_{\mathrm {T}}^{\text {miss}})$	${>}0.5\text {\,rad}$	$\text{ Z }/\gamma ^*\rightarrow \ell \ell$, $\mathrm {W}\text{ Z }$
${\varDelta }\phi ({\vec p}_{\mathrm {T}} ^{\, \ell \ell },{\vec p}_{\mathrm {T}}^{\text {miss}})$	${>}2.6\text {\,rad}$ (omitted)	$\text{ Z }/\gamma ^*\rightarrow \ell \ell$
$|p_{\mathrm {T}} ^\text {miss}-p_{\mathrm {T}} ^{\, \ell \ell }|/p_{\mathrm {T}} ^{\, \ell \ell }$	${<}0.4$ (omitted)	$\text{ Z }/\gamma ^*\rightarrow \ell \ell$
${\varDelta }R_{\ell \ell }$	${<}1.8$ (omitted)	$\mathrm {W}\mathrm {W}$, top quark

## Background estimation

Background contributions are estimated using combined information from simulation and control regions (CRs) in data. The normalizations of the dominant background processes are constrained by using a simultaneous maximum likelihood fit to the SR, as well as to the CRs that are described in this section. The contributions of minor backgrounds in both SR and CRs are predicted from simulation.

### Diboson background

The $\text{ Z } \text{ Z }$ and $\mathrm {W}\text{ Z }$ processes contribute to the SR via the ${\text{ Z } \text{ Z } \rightarrow \ell \ell \nu \nu }$ and ${\mathrm {W}\text{ Z } \rightarrow \ell \nu \ell \ell }$ decay modes, respectively, where the decay products of one boson are not detected. The background estimate for these processes is improved by selecting CRs with alternative decay modes that not only provide a normalization based on CRs in data, but also probe the lost-boson $p_{\mathrm {T}}$ distribution, which is expected to be independent of the decay mode. In this way, the $p_{\mathrm {T}} ^\text {miss}$ spectra of these processes are constrained with respect to their theoretical predictions.

The ability of the simulation to correctly model the lost-boson rapidity is important, as the SR rapidity acceptance of the lost boson is necessarily larger than the rapidity acceptance of the proxy boson in each CR, due to the fact that the visible decay products of the proxy boson in the CR must be inside the detector acceptance. The impact of possible data-to-simulation discrepancies in the high-rapidity portion of diboson background in the SR is suppressed by the fact that, as measured in simulation, the majority of the $\mathrm {W}\text{ Z }$ and $\text{ Z } \text{ Z }$ contamination in the SR is comprised of events where the lost boson is within the rapidity range of the CRs. In addition, the proxy boson rapidity distributions in the CRs (or its visible lepton, in the case of the $\mathrm {W}\text{ Z }$ CR) show a good agreement between data and simulation.

#### The WZ control region

The $\mathrm {W}\text{ Z }$ control region is formed from events with three well-reconstructed charged leptons. In this case, the CR is populated by events with the same decay mode as the SR, but no leptons are lost to identification or acceptance requirements. A $\text{ Z }$ boson candidate is selected in the same manner as for the SR, and an additional electron or muon, with identical quality requirements as applied to the leptons in the SR, is required. To enhance the purity of the $\mathrm {W}\text{ Z }$ selection, $p_{\mathrm {T}} ^\text {miss}$ of at least 30$\,\text {GeV}$ is required, the invariant mass of three leptons is required to be larger than $100\,\text {GeV}$, and the invariant masses of all opposite-sign, same-flavor lepton pairs are required to be larger than $4\,\text {GeV}$. Backgrounds in this CR are similar to those in the SR, with a sizeable nonprompt background from the DY+jets process, where a jet is misidentified as a lepton. All background estimates for this CR are taken from simulation.

The $\mathrm {W}$ boson $p_{\mathrm {T}}$ (“emulated $p_{\mathrm {T}} ^\text {miss}$ ”) is estimated by calculating the vectorial sum of the ${\vec p}_{\mathrm {T}}^{\text {miss}}$ vector and the transverse momentum vector (${\vec p}_{\mathrm {T}}$) of the third charged lepton. In simulation, the majority (over $70\%$) of $\mathrm {W}\text{ Z }$ background contamination in the signal region originates from events where over $90\%$ of the $\mathrm {W}$ boson transverse momentum is carried by one or more neutrinos from the $\mathrm {W}$ boson decay. Thus, the majority of the W boson rapidity distribution in the SR is central, although it is less central than in the WZ CR. Neither the SR nor the $\mathrm {W}\text{ Z }$ CR topology can probe the W boson rapidity directly. However, for the $\mathrm {W}\text{ Z }$ CR, good agreement between data and simulation in the third lepton pseudorapidity distributions is observed.

A minor source of WZ background contamination in the SR originates from events where the visible lepton from a $\mathrm {W}$ boson decay failed identification requirements. Data-to-simulation discrepancies in this contribution would also manifest in the measured $\mathrm {W}\text{ Z }$ CR $p_{\mathrm {T}} ^\text {miss}$ distribution, for which no such mismodeling effects are evident.

**Fig. 2:**
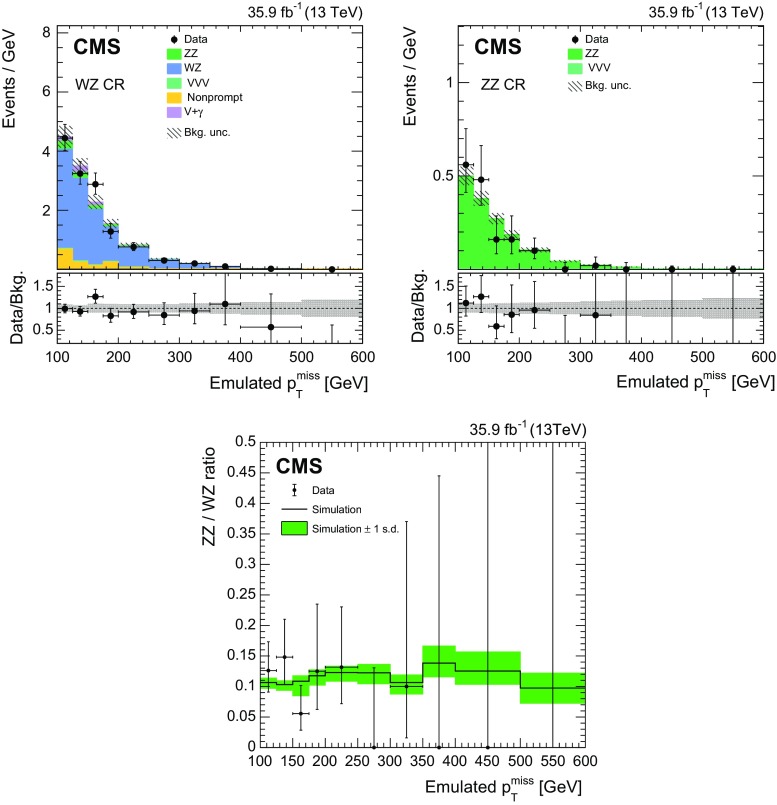
Emulated $p_{\mathrm {T}} ^\text {miss}$ distribution in data and simulation for the $\mathrm {W}\text{ Z } \rightarrow 3\ell \nu$ (upper left) and $\text{ Z } \text{ Z } \rightarrow 4\ell$ (upper right) CRs, and the ratio between both distributions (lower). No events are observed with emulated $p_{\mathrm {T}} ^\text {miss} >500\,\text {GeV}$ in either channel. Uncertainty bands correspond to the combined statistical and systematic components

Using the emulated $p_{\mathrm {T}} ^\text {miss}$ in place of the reconstructed $p_{\mathrm {T}} ^\text {miss}$, the same selection is applied as for the SR. However, since there is no danger of CR contamination from $\mathrm {W}\text{ Z } \rightarrow \tau \nu \ell \ell$ or top quark backgrounds, no veto on additional $\tau _\mathrm {h}$ or b jet candidates is applied. The resulting emulated $p_{\mathrm {T}} ^\text {miss}$ spectrum is shown in Fig. 2 (upper left).

#### The ZZ control region

The $\text{ Z } \text{ Z }$ control region is formed from events with four well-reconstructed charged leptons. In addition to a signal-like $\text{ Z }$ boson candidate, a second $\text{ Z }$ boson candidate is required, the constituents of which only need to pass relaxed lepton quality requirements. This choice reflects the very high purity of the four-lepton selection. For both candidates, the same $\text{ Z }$ boson mass constraint as in the SR is applied. Backgrounds, dominated by triboson processes, are almost negligible in this CR and are taken from simulation.

Similar to the $\mathrm {W}\text{ Z }$ case, the emulated $p_{\mathrm {T}} ^\text {miss}$ is calculated as the vectorial sum of the ${\vec p}_{\mathrm {T}}^{\text {miss}}$ and the ${\vec p}_{\mathrm {T}}$ of the $\text{ Z }$ boson with the larger mass difference to the nominal value of $m_{\text{ Z }}$ of the two identified in the event. The choice of which $\text{ Z }$ boson to use as a proxy for an invisibly decaying one does not significantly alter the emulated $p_{\mathrm {T}} ^\text {miss}$ spectrum. In this CR, the rapidity of the proxy boson is observable, for which good agreement between data and simulation is found.

The same selection as in the SR is then applied using the emulated $p_{\mathrm {T}} ^\text {miss}$ in place of the reconstructed $p_{\mathrm {T}} ^\text {miss}$, with the exception of the $\tau$ lepton and b jet vetoes. The resulting emulated $p_{\mathrm {T}} ^\text {miss}$ spectrum is shown in Fig. 2 (upper right).

#### The VV ratio constraints

Due to a limited event count in the $\text{ Z } \text{ Z }$ control region, the normalizations of the $\mathrm {W}\text{ Z }$ and $\text{ Z } \text{ Z }$ processes in the $\mathrm {W}\text{ Z }$ and $\text{ Z } \text{ Z }$ CRs and the SR are controlled by a single free parameter in the maximum likelihood fit, with their relative normalizations fixed by the theoretical predictions for the $\mathrm {W}\text{ Z }$ and $\text{ Z } \text{ Z }$ processes in each $p_{\mathrm {T}} ^\text {miss}$ bin. The predictions for these processes are obtained from fully reconstructed simulated events generated as described in Sect. 5 with the following additional higher-order corrections applied:

- a constant (approximately $10\%$) correction for the $\mathrm {W}\text{ Z }$ cross section from NLO to NNLO in QCD calculations [76];
- a constant (approximately $3\%$) correction for the $\mathrm {W}\text{ Z }$ cross section from LO to NLO in electroweak (EW) calculations, considering also photon-quark initial states, according to Ref. [77];
- a ${\varDelta }\phi (\text{ Z },\text{ Z })$-dependent correction, varying in magnitude up to $15\%$, to $\text{ Z } \text{ Z }$ production cross section from NLO to next-to-next-to-leading order (NNLO) in QCD calculations [78];
- a $p_{\mathrm {T}}$-dependent correction, varying in magnitude up to $20\%$ at high $p_{\mathrm {T}} ^\text {miss}$, to the $\text{ Z } \text{ Z }$ cross section from LO to NLO in EW calculations, following Refs. [77, 79, 80], which is the dominant correction in the signal region.

We use the product of the above NLO EW corrections and the inclusive NLO QCD corrections [81] as an estimate of the missing $\text {NLO EW}\times \text {NLO QCD}$ contribution, which is not used as a correction, but rather assigned as an uncertainty. The uncertainties in the $\mathrm {W}\text{ Z }$ and $\text{ Z } \text{ Z }$ EW corrections are assumed to be anticorrelated as a conservative measure. The uncertainty associated with the NNLO QCD corrections for both processes is represented by the QCD scale variation uncertainties evaluated on the NLO QCD simulation sample for the respective process, as described in Sect. 10. Figure 2 (lower) shows the ratio of $\text{ Z } \text{ Z }$ to $\mathrm {W}\text{ Z }$ CR yields per $p_{\mathrm {T}} ^\text {miss}$ bin, which probes the validity of taking the relative normalizations from simulation. Good agreement is observed between data and simulation.

### Nonresonant backgrounds

The contribution of the nonresonant flavor-symmetric backgrounds is estimated from a CR based on events with two leptons of different flavor ($\mathrm {e}^{\pm }\mathrm {\mu }^{\mp }$) that pass all other analysis selections. Nonresonant background (NRB) consists mainly of leptonic $\mathrm {W}$ boson decays in ${\text{ t }\overline{\text{ t }}}$, $\text{ t } \mathrm {W}$, and $\mathrm {W}\mathrm {W}$ events, where the dilepton mass happens to fall inside the $\text{ Z }$ boson mass window. Small contributions from single top quark events produced via *s*- and *t*-channel processes, and $\text{ Z } \rightarrow \tau \tau$ events in which $\tau$ leptons decay into light leptons and neutrinos are also considered in the NRB estimation.

The method assumes lepton flavor symmetry in the final states of these processes. Since the leptonic decay branching fraction to the $\mathrm {e}\mathrm {e}$, $\mu \mu$, and $\mathrm {e}\mu$ final states from NRB are 1:1:2, the $\mathrm {e}\mu$ events selected inside the $\text{ Z }$ boson mass window can be extrapolated to the $\mathrm {e}\mathrm {e}$ and $\mu \mu$ channels. To account for differences in efficiency for electrons and muons, a correction factor $k_{\mathrm {e}\mathrm {e}}$ is derived by comparing the NRB yields for the $\mathrm {e}\mathrm {e}$ and $\mu \mu$ channels:

$$ k_{\mathrm {e}\mathrm {e}} = \frac{\epsilon _{\mathrm {e}}}{\epsilon _{\mu }} = \sqrt{\frac{N^{\mathrm {e}\mathrm {e}}_\mathrm {NRB}}{N^{\mu \mu }_\mathrm {NRB}}} $$

under the assumption that there are no efficiency correlations between the two leptons. In simulation, $k_{\mathrm {e}\mathrm {e}}$ is found to be about 0.88 for the final selection. With this correction factor, the relation between the NRB yields in the SR and CR is:

$$\begin{aligned} N^{\ell \ell }_\mathrm {NRB} = \frac{1}{2} \left( k_{\mathrm {e}\mathrm {e}} + \frac{1}{k_{\mathrm {e}\mathrm {e}}} \right) N^{\mathrm {e}\mu }_\mathrm {NRB}. \end{aligned}$$

The ratio of the NRB contributions in the SR and CR is fixed by this relation. Their normalization is controlled by a common scaling parameter that is left to float in the maximum likelihood fit. Perturbations in the predicted transfer factor due to data-to-simulation discrepancies in $k_{\mathrm {e}\mathrm {e}}$ are suppressed upon summing the $\mathrm {e}\mathrm {e}+ \mu \mu$ channels. The uncertainty in the transfer factor is set conservatively to 20%.

### The Drell–Yan background

The DY background is dominant in the region of low $p_{\mathrm {T}} ^\text {miss}$. This process does not produce undetectable particles, therefore any nonzero $p_{\mathrm {T}} ^\text {miss}$ arises from the limited detector acceptance and mismeasurement. The estimation of this background uses simulated DY events, for which the normalization is taken from data in a sideband CR of $50 \le p_{\mathrm {T}} ^\text {miss} \le 100 \,\text {GeV}$, with all other selections applied. In two CRs where a larger DY background contribution is expected, regions with inverted selections on ${\varDelta }\phi ({\vec p}_{\mathrm {T}} ^{\, \ell \ell },{\vec p}_{\mathrm {T}}^{\text {miss}})$ and on $|p_{\mathrm {T}} ^\text {miss}-p_{\mathrm {T}} ^{\, \ell \ell }|/p_{\mathrm {T}} ^{\, \ell \ell }$, the simulation is found to model the data well. The sideband CR is included in the maximum likelihood fit, for which the normalization factor is found to be consistent with unity, and a 100% uncertainty is assigned to the resulting DY estimate in order to cover the extrapolation from this CR to the SR. This uncertainty has little effect on the results owing to the small overall contribution from the DY process in the high-$p_{\mathrm {T}} ^\text {miss}$ SR of this analysis.

## Multivariate analysis

**Fig. 3:**
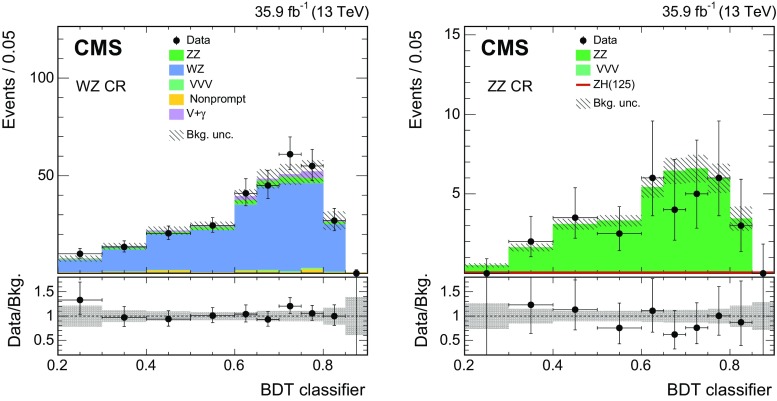
Distribution of the BDT classifier in the diboson CRs: (left) $\mathrm {W}\text{ Z }$ CR; (right) $\text{ Z } \text{ Z }$ CR. Uncertainty bands correspond to the combined statistical and systematic components

For the specific interpretation of this analysis involving invisible decays of the SM (125$\,\text {GeV}$) Higgs boson, a maximum likelihood fit is performed to the spectrum of the BDT classifier values for events satisfying the BDT SR criteria described in Sect. 7, with the classifier value between 0.2 and 1. The CR strategy is identical to that in the $p_{\mathrm {T}} ^\text {miss}$-based analysis, as described in Sect. 8. The three- and four-lepton events shown in Fig. 3 are chosen using the same CR selections as in the $p_{\mathrm {T}} ^\text {miss}$-based analysis.

The multivariate classifier improves the sensitivity of the analysis to the SM H(inv.) model by 10% compared to the $p_{\mathrm {T}} ^\text {miss}$-based analysis. Other than the $p_{\mathrm {T}} ^\text {miss}$ itself, the variables that provide the most discrimination power are the transverse masses of each lepton with respect to the ${\vec p}_{\mathrm {T}}^{\text {miss}}$, along with the azimuthal separation between the ${\vec p}_{\mathrm {T}}^{\text {miss}}$ and the dilepton system momentum. Utilization of this classifier for the other signal models considered in this paper was not pursued, as many of the models’ kinematic distributions can vary considerably over the relevant parameter space.

## Efficiencies and systematic uncertainties

The efficiency for all backgrounds is estimated using simulation. The uncertainties in the yields from missing higher-order corrections in signal as well as $\text{ Z } \text{ Z }$ and $\mathrm {W}\text{ Z }$ background cross sections are evaluated by independently varying up and down the factorization and renormalization scales by a factor of two. The effect of these variations on the yields is between 5 and 10%. For the $\text{ Z } \text{ Z }$ and $\mathrm {W}\text{ Z }$ backgrounds, additional uncertainties related to known higher-order corrections are applied, as discussed in Sect. 8.

For the Higgs boson signal, the PDF and $\alpha _{s}$ uncertainties comprise the cross section normalization uncertainties computed by the LHC Higgs cross section working group [82] and the effect on the signal acceptance of varying the PDFs and $\alpha _{s}$ within their uncertainties [83]. For other signal models, as well as the $\mathrm {W}\text{ Z }$ and $\text{ Z } \text{ Z }$ backgrounds, the effects of the PDF and $\alpha _{s}$ uncertainties in the signal acceptance are taken into account following the PDF4LHC prescription [83]. The PDF and $\alpha _{s}$ uncertainties on these processes are found to be about 1–2%.

The efficiencies for triggering on, reconstructing, and identifying isolated leptons are obtained from simulation, and corrected with scale factors determined via a “tag-and-probe” technique [84] applied to $\text{ Z } \rightarrow \ell ^+\ell ^-$ events in data. The associated uncertainty is about 1–2% per lepton. An additional 3% uncertainty associated with the $\mathrm {W}\text{ Z } \rightarrow \ell \nu \ell \ell$ events, where the reconstructed lepton from the $\mathrm {W}$ boson decay fails identification, is also included.

In order to reproduce b tagging efficiencies and light-flavor jet mistag rates observed in data, an event-by-event reweighting using data-to-simulation scale factors [72] is applied to simulated events. The uncertainty associated with this procedure is obtained by varying the event-by-event weight by ${\pm }1$ standard deviation (s.d.). The impact on the final yields due to the b tagging efficiency and mistag rate uncertainties is around 1% for both signal and background.

The impacts of the jet energy scale and resolution uncertainties are estimated by shifting reconstructed jet energies in simulation by ${\pm }1$ s.d., and each is found to have an effect of about 2% on the yields of the simulated processes after all selections are applied. The impacts of the electron and muon energy scales are evaluated in the same manner, and have a similar effect. Uncertainties in the $p_{\mathrm {T}} ^\text {miss}$ measurement due to the energy resolution of unclustered PF candidates (i.e., those not associated with an electron, muon, or jet) amount to about 2%.

The uncertainty in the expected yields due to the finite size of the MC samples is considered, and is around 1% for the signal and main backgrounds. The simulated MC samples are reweighted to reproduce the pileup conditions observed in data. The uncertainty related to this procedure is obtained by varying the central value of the estimated inelastic cross section by 5% [85], and is found to be below 1%. The uncertainty assigned to the integrated luminosity measurement is 2.5% [86].

The effect of the systematic uncertainties on the shape of the distribution of the discriminating variable ($p_{\mathrm {T}} ^\text {miss}$ or BDT classifier) is taken into account by varying the value of the quantity associated with the uncertainty, and observing the resulting variations in the individual bins of $p_{\mathrm {T}} ^\text {miss}$.

In addition to all of the sources of systematic uncertainty in the $p_{\mathrm {T}} ^\text {miss}$-based analysis, the following systematic uncertainties in the BDT-based analysis affect the BDT classifier shape. The most important sources of uncertainty in the BDT classifier shape are the lepton energy scale and $p_{\mathrm {T}} ^\text {miss}$ uncertainties; their impact on the signal ($\mathrm {W}\text{ Z }$ and $\text{ Z } \text{ Z }$ backgrounds) amounts to about 2 (6)% and translates into an additional 2% uncertainty in the expected limit on the H(inv.) branching fraction.

All these sources of uncertainty are summarized in Table 2. The combined uncertainty in the signal efficiency and acceptance is estimated to be about 5% and is dominated by the theoretical uncertainty due to missing higher-order corrections and PDF uncertainties. The total uncertainty in the background estimations in the signal region is about 15%, dominated by the theoretical uncertainties in the $\text{ Z } \text{ Z }$ and $\mathrm {W}\text{ Z }$ process description.

**Table 2:** Summary of the systematic uncertainties for the $p_{\mathrm {T}} ^\text {miss}$- and BDT-based analyses. Each uncertainty represents the variation of the relative yields of the processes in the SR. Each uncertainty is fully correlated across processes to which it contributes, including those processes that are also present in CRs. The symbol “—” indicates that the systematic uncertainty does not contribute or is deemed negligible. For minor backgrounds, systematic uncertainties are omitted because of the smallness of their contribution. For shape uncertainties (indicated with a *), the numbers correspond to the overall effect of the shape variation on the yield or acceptance. The impact on the expected upper limit for the signal strength, i.e., the relative decrease in the median expected upper limit for the signal strength upon removing the nuisance term, is evaluated with respect to the SM H(inv.) signal and presented in the last column. In this column the number in parentheses shows the impact on the BDT-based analysis, if different from that for the $p_{\mathrm {T}} ^\text {miss}$-based analysis. The last part of the table provides the additional uncertainties in the BDT-based analysis

Source of uncertainty	Effect (%)					Impact on the exp. limit (%)
	Signal	$\text{ Z } \text{ Z }$	$\mathrm {W}\text{ Z }$	NRB	DY	
* VV EW corrections	—	10	$-4$	—	—	14 (12)
* Renorm./fact. scales, VV	—	9	4	—	—	2 (1)
* Renorm./fact. scales, ZH	3.5	—	—	—	—	
* Renorm./fact. scales, DM	5	—	—	—	—	
* PDF, WZ background	—	—	1.5	—	—	
* PDF, ZZ background	—	1.5	—	—	—	
* PDF, Higgs boson signal	1.5	—	—	—	—	
* PDF, DM signal	1–2	—	—	—	—	
* MC sample size, NRB	—	—	—	5	—	1
* MC sample size, DY	—	—	—	—	30	
* MC sample size, $\text{ Z } \text{ Z }$	—	0.1	—	—	—	
* MC sample size, $\mathrm {W}\text{ Z }$	—	—	2	—	—	
* MC sample size, ZH	1	—	—	—	—	
* MC sample size, DM	3	—	—	—	—	
NRB extrapolation to the SR	—	—	—	20	—	<1
DY extrapolation to the SR	—	—	—	—	100	<1
Lepton efficiency (WZ CR)	—	—	3	—	—	<1
Nonprompt bkg. (WZ CR)	—	—	—	—	30	<1
Integrated luminosity	2.5					<1
* Electron efficiency	1.5					1 (<1)
* Muon efficiency	1					
* Electron energy scale	1–2					
* Muon energy scale	1–2					
* Jet energy scale	1–3 (typically anticorrelated w/ yield)					
* Jet energy resolution	1 (typically anticorr.)					
* Unclustered energy ($p_{\mathrm {T}} ^\text {miss}$)	1–4 (typically anticorr.), strong in DY					
* Pileup	1 (typically anticorrelated)					
* b tagging eff. & mistag rate	1					
* BDT: electron energy scale	1.1	2.9	2.6	—	—	— (2)
* BDT: muon energy scale	1.5	4.3	2.7	—	—	
* BDT: $p_{\mathrm {T}} ^\text {miss}$ scale	1.0	3.2	4.1	—	—	

## Results

**Fig. 4:**
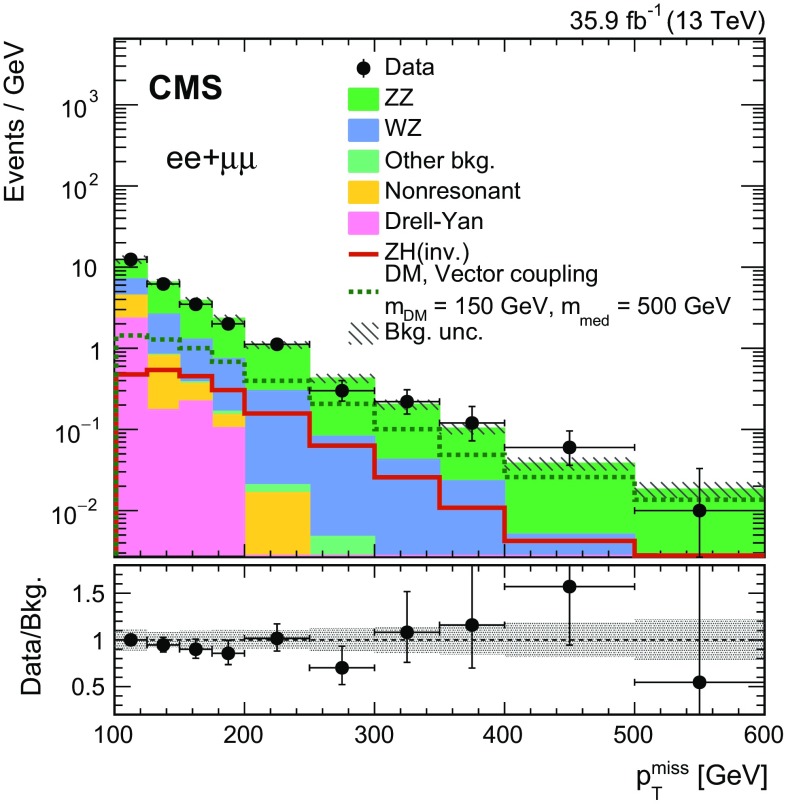
Distribution of the $p_{\mathrm {T}} ^\text {miss}$ in the combination of the $\mathrm {e}\mathrm {e}$ and $\mu \mu$ channels after the full selection. The last bin also includes any events with $p_{\mathrm {T}} ^\text {miss} >600\,\text {GeV}$. The uncertainty band includes both statistical and systematic components. The $\text{ Z } \text{ H } (\text {inv.})$ signal normalization assumes SM production rates and the branching fraction $\mathcal {B}(\text{ H } \rightarrow \text {inv.}) = 1$

The numbers of observed and expected events for the $p_{\mathrm {T}} ^\text {miss}$-based analysis are shown in Table 3. There is no significant difference between the dielectron and dimuon channels in terms of signal-to-background ratio, and hence both are treated together when obtaining the final results. The observed number of events in the $\mathrm {e}\mathrm {e}$ ($\mu \mu$) channel is 292 (406), and the number of events expected from simulation is $301\pm 23$ ($391\pm 26$). Figure 4 shows the $p_{\mathrm {T}} ^\text {miss}$ distribution in the $\mathrm {e}\mathrm {e}+\mu \mu$ channel in the SR. The total background estimates and the observed numbers of events in each $p_{\mathrm {T}} ^\text {miss}$ bin are listed in Table 4, for both a combined background-only fit to the SR and the CRs, as well as for a fit to the CRs only. The latter results can be used in conjunction with the SR bin correlation matrix presented in the supplemental material 1 to recast these results in the simplified likelihood framework [87].

**Table 3:** Signal predictions, post-fit background estimates, and observed numbers of events in the $p_{\mathrm {T}} ^\text {miss}$-based analysis. The combined statistical and systematic uncertainties are reported

Process	$\mathrm {e}\mathrm {e}+ \mu \mu$
qqZH(inv.)	$158.6 \pm 5.4\phantom {0}\phantom {0}$
$\ \ m_{\text{ H }}=125 \,\text {GeV}$, $\mathcal {B}({\text{ H } \rightarrow \text {inv.}}) = 1$	
ggZH(inv.)	$42.7 \pm 4.9\phantom {0}$
$\ \ m_{\text{ H }}=125 \,\text {GeV}$, $\mathcal {B}({\text{ H } \rightarrow \text {inv.}}) = 1$	
DM, vector mediator	$98.8 \pm 3.9\phantom {0}$
$\ \ m_\text {med}=500 \,\text {GeV}$, $m_\mathrm {DM} = 150 \,\text {GeV}$	
DM, axial-vector mediator	$65.5 \pm 2.6\phantom {0}$
$\ \ m_\text {med}=500 \,\text {GeV}$, $m_\mathrm {DM} = 150\,\text {GeV}$	
ZZ	$379.8 \pm 9.4\phantom {0}\phantom {0}$
WZ	$162.5 \pm 6.8\phantom {0}\phantom {0}$
Nonresonant bkg.	$75 \pm 15$
Drell–Yan	$72 \pm 29$
Other bkg.	$2.6 \pm 0.2$
Total bkg.	$692 \pm 35\phantom {0}$
Data	698

**Table 4:** Expected event yields in each $p_{\mathrm {T}} ^\text {miss}$ bin for the sum of background processes in the SR. The background yields and their corresponding uncertainties are obtained after performing a fit to data. Two sets of background yields are reported: one from a background-only fit to data in both the SR and the CRs, and one from a fit to data in all CRs, but excluding data in the SR. The observed numbers of events in each bin are also included

$p_{\mathrm {T}} ^\text {miss}$ bin ($\text {GeV}$ )	Observed events	Total background prediction	
		SR+CR fit	CR-only fit
$100 \le p_{\mathrm {T}} ^\text {miss} < 125$	311	300, 18	256,32
$125 \le p_{\mathrm {T}} ^\text {miss} < 150$	155	155.0, 7.0	150,12
$150 \le p_{\mathrm {T}} ^\text {miss} < 175$	87	90.8, 4.6	86.9,8.4
$175 \le p_{\mathrm {T}} ^\text {miss} < 200$	50	54.7, 3.1	52.7,5.3
$200 \le p_{\mathrm {T}} ^\text {miss} < 250$	56	51.3, 2.9	50.2,4.9
$250 \le p_{\mathrm {T}} ^\text {miss} < 300$	15	19.7, 1.4	19.4,2.2
$300 \le p_{\mathrm {T}} ^\text {miss} < 350$	11	9.64, 0.80	9.4,1.2
$350 \le p_{\mathrm {T}} ^\text {miss} < 400$	6	4.73, 0.47	4.58,0.66
$400 \le p_{\mathrm {T}} ^\text {miss} < 500$	6	3.44, 0.39	3.31,0.54
$p_{\mathrm {T}} ^\text {miss} \ge 500$	1	1.63, 0.24	1.57,0.33

No deviation from the SM background expectation is found. Upper limits on the contribution of events from new physics are computed by using the modified frequentist approach $CL_s$ [88, 89] based on asymptotic formulas [90, 91], via a simultaneous maximum likelihood fit to the SR and the CRs. The expected numbers of background events and signal events, scaled by a signal strength modifier, are combined in a profile likelihood test statistic, in which the systematic uncertainties are incorporated as nuisance parameters. For the dominant backgrounds in the SR, additional parameters are introduced to link the background expectations in the SR to their respective contributions in the CRs discussed in Sect. 8. To compute limits in all models, a binned likelihood test statistic is employed, based on the $p_{\mathrm {T}} ^\text {miss}$ distribution in Fig. 4 and also on the BDT classifier distribution in the case of invisible decays of the SM Higgs boson.

### Dark matter interpretation

**Fig. 5:**
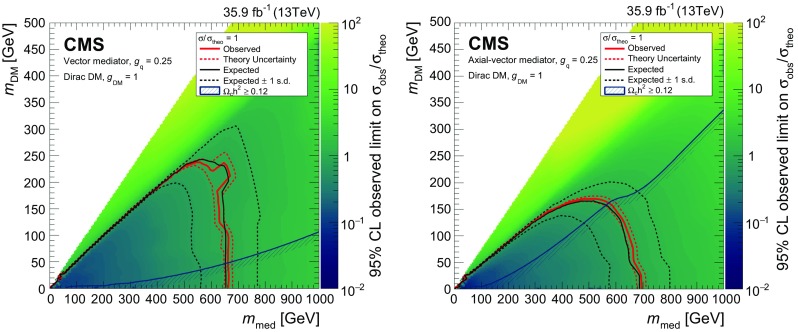
The 95% CL expected and observed limits on $\sigma _\text {obs}/\sigma _\text {theo}$ for the vector (left) and axial-vector (right) mediators with $g_{\mathrm {q}}=0.25$ and $g_\mathrm {DM} = 1$. Limits are not shown for far off-shell ($2m_\mathrm {DM} > 1.5 m_\text {med}$) regions of the parameter space

**Fig. 6:**
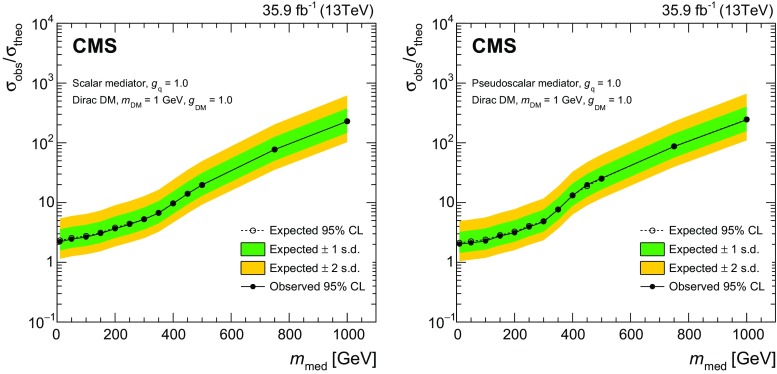
The 95% CL expected and observed limits on $\sigma _\text {obs}/\sigma _\text {theo}$ for the scalar (left) and pseudoscalar (right) mediated DM scenario with $g_{\mathrm {q}}=g_\mathrm {DM}=1$. The limits are parameterized as a function of mediator mass $m_\text {med}$ for a fixed dark matter mass $m_\mathrm {DM}=1\,\text {GeV}$

**Fig. 7:**
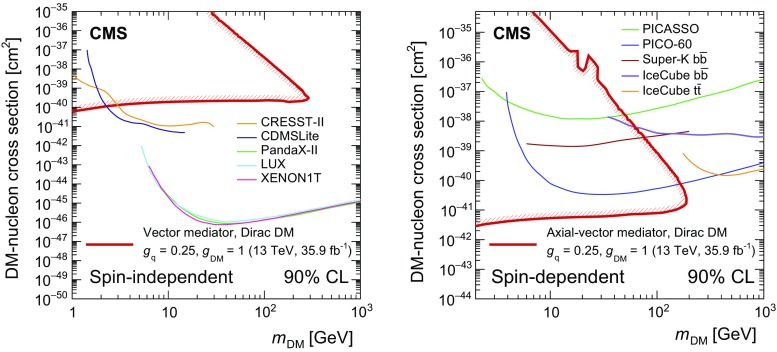
Observed 90% CL limits on the DM-nucleon scattering cross sections in both spin-independent (left) and spin-dependent (right) cases, assuming a mediator-quark coupling constant $g_{\mathrm {q}} = 0.25$ and mediator-DM coupling constant $g_\mathrm {DM} = 1$. Limits from the CRESST-II [92], CDMSLite [93], PandaX-II [94], LUX [95], and XENON1T [96] experiments are shown for the spin-independent case (vector couplings). Limits from the PICASSO [97], PICO-60 [98], Super-Kamiokande [99], and IceCube [100, 101] experiments are shown for the spin-dependent case (axial-vector couplings)

Figure 5 shows the 95% CL expected and observed limits for vector and axial-vector scenarios with couplings $g_{\mathrm {q}}=0.25$, $g_\mathrm {DM}=1$. Figure 6 shows the 95% CL expected and observed limits for couplings $g_{\mathrm {q}}=g_\mathrm {DM}= 1$ in the scalar and pseudoscalar scenarios. In Fig. 7, limits on the DM-nucleon scattering cross section are set at 90% CL as a function of the DM particle mass and compared to selected results from direct detection experiments. Both spin-dependent and spin-independent cases are considered. In both cases, couplings $g_{\mathrm {q}}=0.25$ and $g_\mathrm {DM}=1$ are used.

### Limits on invisible Higgs boson decays

**Fig. 8:**
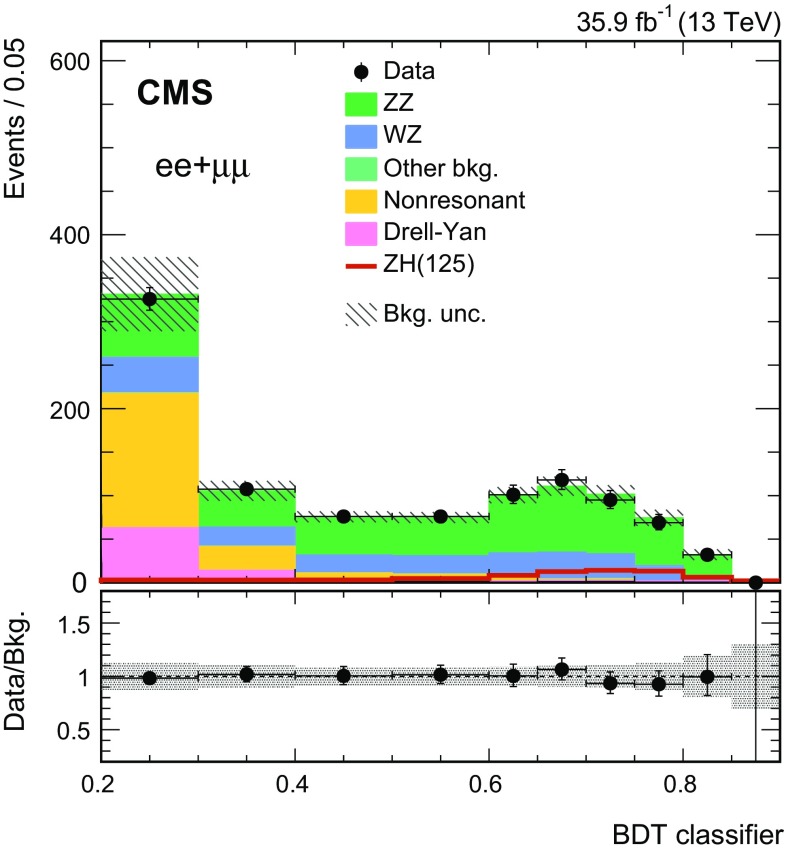
Post-fit distribution of the BDT classifier in the multivariate analysis signal region for the SM H(inv.) decay hypothesis with $\mathcal {B}(\text{ H } \rightarrow \text {inv.}) = 100\%$. Uncertainty bands correspond to the combined statistical and systematic components

**Fig. 9:**
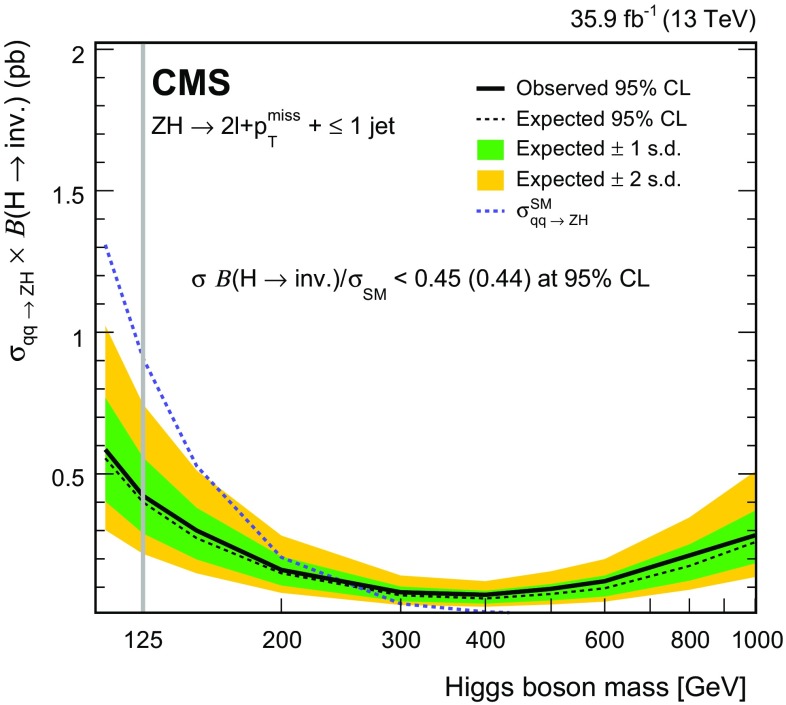
Expected and observed 95% CL upper limits on the product of the production cross section and the branching fraction, $\sigma_{\mathrm{qq}\to\mathrm{ZH}}\mathcal{B}(\mathrm{H}\to\text{inv.})$, as a function of the SM-like Higgs boson mass. The limits consider only quark-induced Higgs boson production. In addition, for the SM (125$\,\text {GeV}$) Higgs boson, the limit on branching fraction assuming SM production rate (considering also gluon fusion) is presented. The vertical gray line indicates that the result at $m_{\text{H }}=125\,\text {GeV}$ should not be read from the plot, as the gluon contribution is known for that point

Upper limits are derived for the Higgs boson production cross section using the same $p_{\mathrm {T}} ^\text {miss}$-shape analysis as for the DM model. In addition, for $m_{\text{H }}= 125\,\text {GeV}$, a shape analysis using the multivariate classifier distribution, as described in Sect. 9, is performed. The resulting post-fit signal region is shown in Fig. 8. The 95% CL expected and observed upper limits on the product of the production cross section and the branching fraction, $\sigma _{\text{Z} \text{H}} \, \mathcal {B}(\text{ H } \rightarrow \text {inv.})$, computed with the asymptotic $CL_s$ method are shown as a function of the SM-like Higgs boson mass in Fig. 9 for the $p_{\mathrm {T}} ^\text {miss}$-shape analysis. For $m_{\text{ H }}= 125\,\text {GeV}$, the search can be interpreted as an upper limit on $\mathcal {B}(\text{ H } \rightarrow \text {inv.})$ assuming the SM production rate of a Higgs boson in association with a $\text{ Z }$ boson. Assuming the SM production rate, the 95% observed (expected) CL upper limit on $\mathcal {B}(\text{ H } \rightarrow \text {inv.})$ is 0.45 (0.44) using the $p_{\mathrm {T}} ^\text {miss}$-shape analysis, and 0.40 (0.42) using the multivariate analysis. The $\mathrm {gg} \rightarrow \text{ Z } (\ell \ell )\text{ H }$ process is considered only for the 125 GeV mass point, and only when interpreting the result as a limit on branching fraction. For SM-like Higgs production, considering only the $\mathrm {qq}\rightarrow \text{ Z } (\ell \ell )\text{ H }$ process, upper limits on $\mathcal {B}(\text{ H } \rightarrow \text {inv.})$ are presented as a function of $m_{\text{ H }}$ in the supplemental material 1.

### Unparticle interpretation

**Fig. 10:**
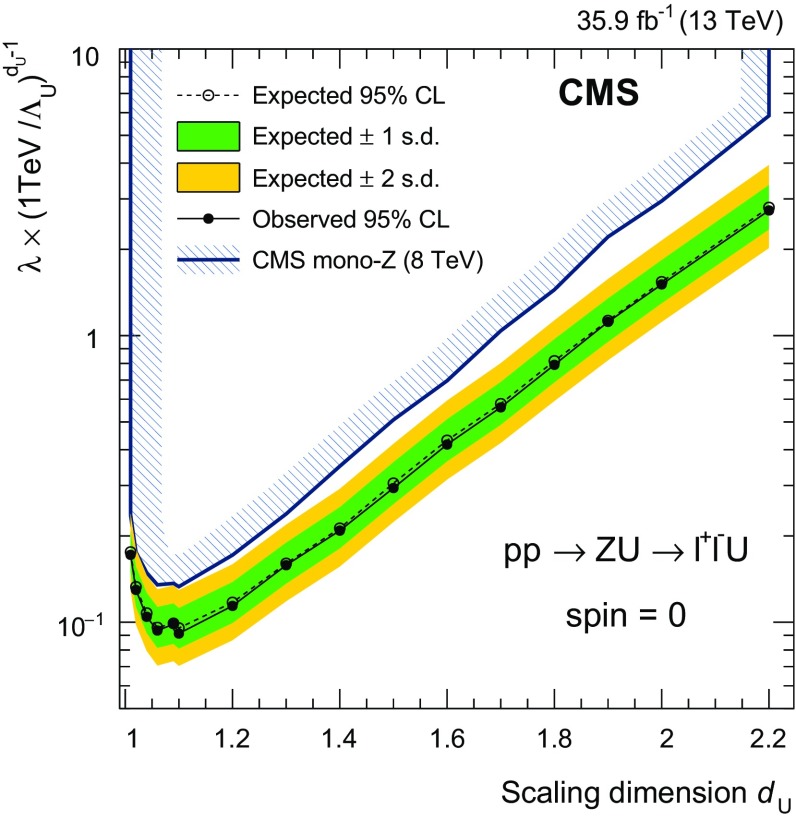
The 95% CL upper limits on the Wilson coefficient $\lambda / {\varLambda }_{\textsf {U}} ^{d_{\textsf {U}}-1}$ of the unparticle-quark coupling operator. The results from an earlier CMS search in the same final state [39] are shown for comparison

In the unparticle scenario, a shape analysis of the $p_{\mathrm {T}} ^\text {miss}$ spectrum is performed. Upper limits are set at 95% CL on the Wilson coefficient $\lambda / {\varLambda }_{\textsf {U}} ^{d_{\textsf {U}}-1}$ of the unparticle-quark coupling operator, and are shown in Fig. 10 as a function of the scaling dimension $d_\textsf {U}$.

### The ADD interpretation

**Fig. 11:**
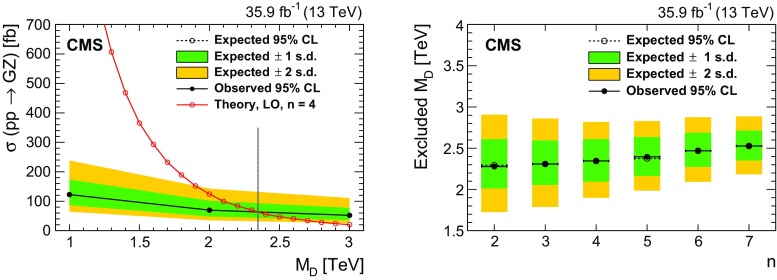
Expected and observed 95% CL cross section exclusion limits for the example case $n=4$ in the ADD scenario (left) and exclusion limits on $M_\mathrm {D}$ for different values of *n* (right). In both plots, the markers for the expected exclusion are obscured by the close overlap with those for the observed exclusion. The red solid line in the left plot shows the theoretical cross section for the case $n = 4$. Cross sections are calculated in the fiducial phase space of $p_{\mathrm {T}} ^\mathrm{G} > 50\,\text {GeV}$. The vertical line in the left plot shows the projection onto the $M_\mathrm {D}$ axis of the intersection of the theory curve with the expected and observed exclusion limits

In the framework of the ADD model of large extra dimensions, we calculate limits depending on the number of extra dimensions *n* and the fundamental Planck scale $M_\mathrm {D}$. For each value of *n*, cross section limits are calculated as a function of $M_\mathrm {D}$. By finding the intersection between the theory cross section line, calculated in the fiducial phase space of the graviton transverse momentum $p_{\mathrm {T}} ^\mathrm{G} > 50\,\text {GeV}$, with the observed and expected excluded cross sections, and projecting that point onto the $M_\mathrm {D}$ axis, we find limits on $M_\mathrm {D}$ as a function of *n*, as shown in Fig. 11.

The observed and expected exclusion of $M_\mathrm {D}$ ranges between 2.3 and 2.5$\,\text {TeV}$ for *n* between 2 and 7, at 95% CL.

## Summary

A search for new physics in events with a leptonically decaying $\text{ Z }$ boson and large missing transverse momentum has been presented. The search is based on a data set of proton-proton collisions collected with the CMS experiment in 2016, corresponding to an integrated luminosity of $35.9 \pm 0.9{\,\text {fb}^{-1}}$ at $\sqrt{s} = 13\,\text {TeV}$. No evidence for physics beyond the standard model is found. Compared to the previous search in the same final state [31], the exclusion limits on dark matter and mediator masses are significantly extended for spin-1 mediators in the simplified model interpretation, and exclusion limits for unparticles are also extended. Results for dark matter production via spin-0 mediators in the simplified model interpretation, as well as graviton emission in a model with large extra dimensions, are presented in this final state for the first time. In the case of invisible decays of a standard-model-like Higgs boson, the upper limit of 40% on their branching fraction is set at 95% confidence level, using data not included in the previously published combined analysis [36].

## Electronic supplementary material

Below is the link to the electronic supplementary material.

Supplementary material 1 (pdf 207 KB)

## References

[1] Hinshaw G (2013). Nine-year Wilkinson Microwave Anisotropy Probe (WMAP) observations: cosmological parameter results. Astrophys. J. Suppl..

[2] P. Cushman et al., Snowmass CF1 summary: WIMP dark matter direct detection (2013). arXiv:1310.8327

[3] J. Buckley et al., Indirect dark matter detection CF2 working group summary (2013). arXiv:1310.7040

[4] Beltran M (2010). Maverick dark matter at colliders. JHEP.

[5] Goodman J (2011). Constraints on light Majorana dark matter from colliders. Phys. Lett. B.

[6] Bai Y, Fox PJ, Harnik R (2010). The Tevatron at the frontier of dark matter direct detection. JHEP.

[7] Goodman J (2010). Constraints on dark matter from colliders. Phys. Rev. D.

[8] Fox PJ, Harnik R, Kopp J, Tsai Y (2012). Missing energy signatures of dark matter at the LHC. Phys. Rev. D.

[9] Rajaraman A, Shepherd W, Tait TMP, Wijangco AM (2011). LHC bounds on interactions of dark matter. Phys. Rev. D.

[10] D. Abercrombie et al., Dark matter benchmark models for early LHC Run-2 searches: Report of the ATLAS/CMS dark matter forum (2015). arXiv:1507.00966

[11] Ellis J (2017). TikZ-Feynman: Feynman diagrams with TikZ. Comput. Phys. Commun..

[12] ATLAS Collaboration, Observation of a new particle in the search for the Standard Model Higgs boson with the ATLAS detector at the LHC. Phys. Lett. B **716**, 1 (2012). 10.1016/j.physletb.2012.08.020. arXiv:1207.7214

[13] CMS Collaboration, Observation of a new boson at a mass of 125 GeV with the CMS experiment at the LHC. Phys. Lett. B **716**, 30 (2012). 10.1016/j.physletb.2012.08.021. arXiv:1207.7235

[14] CMS Collaboration, Observation of a new boson with mass near 125 GeV in pp collisions at $$\sqrt{s} = 7$$ and 8TeV. JHEP **06**, 081 (2013). 10.1007/JHEP06(2013)081. arXiv:1303.4571

[15] Ghosh D (2013). Looking for an invisible Higgs signal at the LHC. Phys. Lett. B.

[16] Martin SP, Wells JD (1999). Motivation and detectability of an invisibly decaying Higgs boson at the Fermilab Tevatron. Phys. Rev. D.

[17] Bai Y, Draper P, Shelton J (2012). Measuring the invisible Higgs width at the 7 and 8TeV LHC. JHEP.

[18] Belanger G (2001). The MSSM invisible Higgs in the light of dark matter and g-2. Phys. Lett. B.

[19] Giudice GF, Rattazzi R, Wells JD (2001). Graviscalars from higher dimensional metrics and curvature Higgs mixing. Nucl. Phys. B.

[20] M. Battaglia, D. Dominici, J.F. Gunion, J.D. Wells, The invisible Higgs decay width in the ADD model at the LHC (2004). arXiv:hep-ph/0402062

[21] Baek S, Ko P, Park W-I, Senaha E (2013). Higgs portal vector dark matter : revisited. JHEP.

[22] Djouadi A, Lebedev O, Mambrini Y, Quevillon J (2012). Implications of LHC searches for Higgs-portal dark matter. Phys. Lett. B.

[23] A. Djouadi, A. Falkowski, Y. Mambrini, J. Quevillon, Direct detection of Higgs-portal dark matter at the LHC. Eur. Phys. J. C **73**(6), 2455 (2013). 10.1140/epjc/s10052-013-2455-1. arXiv:1205.3169

[24] Arkani-Hamed N, Dimopoulos S, Dvali GR (1998). The hierarchy problem and new dimensions at a millimeter. Phys. Lett. B.

[25] Arkani-Hamed N, Dimopoulos S, Dvali GR (1999). Phenomenology, astrophysics and cosmology of theories with submillimeter dimensions and TeV scale quantum gravity. Phys. Rev. D.

[26] Han T, Lykken JD, Zhang R-J (1999). On Kaluza-Klein states from large extra dimensions. Phys. Rev. D.

[27] Banks T, Zaks A (1982). On the phase structure of vector-like gauge theories with massless fermions. Nucl. Phys. B.

[28] Kang Z (2015). Upgrading sterile neutrino dark matter to FI$$m$$P using scale invariance. Eur. Phys. J. C.

[29] Rinaldi M, Cognola G, Vanzo L, Zerbini S (2015). Inflation in scale-invariant theories of gravity. Phys. Rev. D.

[30] Cheng H (1988). The possible existence of Weyl’s vector meson. Phys. Rev. Lett..

[31] CMS Collaboration, Search for dark matter and unparticles in events with a Z boson and missing transverse momentum in proton-proton collisions at $$\sqrt{s} = 13\text{TeV}$$. JHEP **03**, 061 (2017). 10.1007/JHEP03(2017)061. arXiv:1701.02042

[32] ATLAS Collaboration, Search for dark matter at $$\sqrt{s}=13\text{ TeV }$$ in final states containing an energetic photon and large missing transverse momentum with the ATLAS detector. Eur. Phys. J. C **77**, 393 (2017). 10.1140/epjc/s10052-017-4965-8. arXiv:1704.03848 10.1140/epjc/s10052-017-4965-8 PMC5515389 28781578

[33] ATLAS Collaboration, Measurement of detector-corrected observables sensitive to the anomalous production of events with jets and large missing transverse momentum in $$pp$$ collisions at $$\sqrt{s} = 13\text{ TeV }$$ using the ATLAS detector (2017). arXiv:1707.03263. (**submitted to EPJC**) 10.1140/epjc/s10052-017-5315-6 PMC6956934 31999280

[34] CMS Collaboration, Search for dark matter produced with an energetic jet or a hadronically decaying W or Z boson at $$ \sqrt{s}=13\text{ TeV }$$. JHEP **07**, 014 (2017). 10.1007/JHEP07(2017)014. arXiv:1703.01651

[35] ATLAS Collaboration, Constraints on new phenomena via Higgs boson couplings and invisible decays with the ATLAS detector. JHEP **11**, 206 (2015). 10.1007/JHEP11(2015)206. arXiv:1509.00672

[36] CMS Collaboration, Searches for invisible decays of the Higgs boson in pp collisions at $$\sqrt{s} = 7$$, 8, and 13TeV. JHEP **02**, 135 (2017). 10.1007/JHEP02(2017)135. arXiv:1610.09218

[37] CMS Collaboration, Search for dark matter, extra dimensions, and unparticles in monojet events in proton-proton collisions at $$\sqrt{s} = 8\text{ TeV }$$. Eur. Phys. J. C **75**, 235 (2015). 10.1140/epjc/s10052-015-3451-4. arXiv:1408.3583 10.1140/epjc/s10052-015-3451-4 PMC4455910 26069461

[38] ATLAS Collaboration, Search for new phenomena in final states with an energetic jet and large missing transverse momentum in $$pp$$ collisions at $$\sqrt{s}=13\text{ TeV }$$ using the ATLAS detector. Phys. Rev. D **94**, 032005 (2016). 10.1103/PhysRevD.94.032005. arXiv:1604.07773

[39] CMS Collaboration, Search for dark matter and unparticles produced in association with a Z boson in proton-proton collisions at $$\sqrt{s} = 8\text{ TeV }$$. Phys. Rev. D **93**, 052011 (2015). 10.1103/PhysRevD.93.052011. arXiv:1511.09375

[40] CMS Collaboration, The CMS trigger system. JINST **12**, P01020 (2017). 10.1088/1748-0221/12/01/P01020. arXiv:1609.02366

[41] CMS Collaboration, The CMS experiment at the CERN LHC. JINST **3**, S08004 (2008). 10.1088/1748-0221/3/08/S08004

[42] Alioli S, Nason P, Oleari C, Re E (2008). NLO vector-boson production matched with shower in POWHEG. JHEP.

[43] Nason P (2004). A new method for combining NLO QCD with shower Monte Carlo algorithms. JHEP.

[44] Frixione S, Nason P, Oleari C (2007). Matching NLO QCD computations with parton shower simulations: the POWHEG method. JHEP.

[45] Alioli S, Nason P, Oleari C, Re E (2010). A general framework for implementing NLO calculations in shower Monte Carlo programs: the POWHEG BOX. JHEP.

[46] Campbell JM, Ellis RK (2010). MCFM for the Tevatron and the LHC. Nucl. Phys. Proc. Suppl..

[47] Alwall J (2014). The automated computation of tree-level and next-to-leading order differential cross sections, and their matching to parton shower simulations. JHEP.

[48] Mattelaer O, Vryonidou E (2015). Dark matter production through loop-induced processes at the LHC: the $$s$$-channel mediator case. Eur. Phys. J. C.

[49] Backović M, Krämer M, Maltoni F, Martini A, Mawatari K, Pellen M (2015). Higher-order QCD predictions for dark matter production at the LHC in simplified models with $$s$$-channel mediators. Eur. Phys. J. C.

[50] Neubert M, Wang J, Zhang C (2016). Higher-order QCD predictions for dark matter production in mono-$$Z$$ searches at the LHC. JHEP.

[51] G. Busoni et al., Recommendations on presenting LHC searches for missing transverse energy signals using simplified $$s$$-channel models of dark matter (2016). arXiv:1603.04156

[52] Sjöstrand T, Mrenna S, Skands PZ (2008). A brief introduction to PYTHIA 8.1. Comput. Phys. Commun..

[53] Ask S (2009). Simulation of $$Z$$ plus graviton/unparticle production at the LHC. Eur. Phys. J. C.

[54] Ask S (2010). Real emission and virtual exchange of gravitons and unparticles in PYTHIA8. Comput. Phys. Commun..

[55] Collaboration CMS (2016). Event generator tunes obtained from underlying event and multiparton scattering measurements. Eur. Phys. J. C.

[56] Alwall J (2008). Comparative study of various algorithms for the merging of parton showers and matrix elements in hadronic collisions. Eur. Phys. J. C.

[57] Frederix R, Frixione S (2012). Merging meets matching in MC@NLO. JHEP.

[58] NNPDF Collaboration, Parton distributions for the LHC Run II. JHEP** 04**, 040 (2015). 10.1007/JHEP04(2015)040. arXiv:1410.8849

[59] GEANT4 Collaboration, GEANT4—a simulation toolkit. Nucl. Instrum. Meth. A **506**, 250 (2003). 10.1016/S0168-9002(03)01368-8

[60] CMS Collaboration, Particle-flow reconstruction and global event description with the CMS detector. JINST **12**, P10003 (2017). 10.1088/1748-0221/12/10/P10003. arXiv:1706.04965

[61] Cacciari M, Salam GP, Soyez G (2008). The anti-$$k_t$$ jet clustering algorithm. JHEP.

[62] Cacciari M, Salam GP, Soyez G (2012). FastJet user manual. Eur. Phys. J. C.

[63] CMS Collaboration, Performance of electron reconstruction and selection with the CMS detector in proton-proton collisions at $$\sqrt{s}= 8\text{ TeV }$$. JINST **10**, 06005 (2015). 10.1088/1748-0221/10/06/P06005. arXiv:1502.02701

[64] CMS Collaboration, The performance of the CMS muon detector in proton-proton collisions at $$\sqrt{s}= 7\text{ TeV }$$ at the LHC. JINST **8**, P11002 (2013). 10.1088/1748-0221/8/11/P11002. arXiv:1306.6905

[65] Cacciari M, Salam GP (2008). Pileup subtraction using jet areas. Phys. Lett. B.

[66] Cacciari M, Salam GP (2006). Dispelling the $$N^{3}$$ myth for the $$k_{t}$$ jet-finder. Phys. Lett. B.

[67] Cacciari GSM, Salam GP (2008). The catchment area of jets. JHEP.

[68] CMS Collaboration, Determination of jet energy calibration and transverse momentum resolution in CMS. JINST **6**, P11002 (2011). 10.1088/1748-0221/6/11/P11002. arXiv:1107.4277

[69] CMS Collaboration, Jet energy scale and resolution in the CMS experiment in pp collisions at 8TeV. JINST **12**, P02014 (2017). 10.1088/1748-0221/12/02/P02014. arXiv:1607.03663

[70] CMS Collaboration, *Performance of missing energy reconstruction in 13TeV pp collision data using the CMS detector*. CMS Physics Analysis Summary CMS-PAS-JME-16-004 (CERN, Geneva, 2016). https://cds.cern.ch/record/2205284

[71] CMS Collaboration, Identification of b-quark jets with the CMS experiment. JINST **8**, 04013 (2013). 10.1088/1748-0221/8/04/P04013. arXiv:1211.4462

[72] CMS Collaboration, *Identification of b quark jets at the CMS Experiment in the LHC Run 2’*. CMS Physics Analysis Summary CMS-PAS-BTV-15-001 (CERN, Geneva, 2015)

[73] CMS Collaboration, Reconstruction and identification of $$\tau $$ lepton decays to hadrons and $$\nu _\tau $$ at CMS. JINST **11**, P01019 (2016). 10.1088/1748-0221/11/01/P01019. arXiv:1510.07488

[74] Particle Data Group, C. Patrignani et al., Review of particle physics. Chin. Phys. C **40**, 100001 (2016). 10.1088/1674-1137/40/10/100001

[75] Collins JC, Soper DE (1977). Angular distribution of dileptons in high-energy hadron collisions. Phys. Rev. D.

[76] Grazzini M, Kallweit S, Rathlev D, Wiesemann M (2016). $$W^{\pm }Z$$ production at hadron colliders in NNLO QCD. Phys. Lett. B.

[77] Baglio J, Ninh LD, Weber MM (2013). Massive gauge boson pair production at the LHC: A next-to-leading order story. Phys. Rev. D.

[78] Grazzini M, Kallweit S, Rathlev D (2015). ZZ production at the LHC: fiducial cross sections and distributions in NNLO QCD. Phys. Lett. B.

[79] Bierweiler A, Kasprzik T, Kahn JH (2013). Vector-boson pair production at the LHC to $$\cal{O}(\alpha ^3)$$ accuracy. JHEP.

[80] Gieseke S, Kasprzik T, Kuhn JH (2014). Vector-boson pair production and electroweak corrections in HERWIG++. Eur. Phys. J. C.

[81] Campbell JM, Ellis RK, Williams C (2011). Vector boson pair production at the LHC. JHEP.

[82] LHC Higgs Cross Section Working Group, Handbook of LHC Higgs cross sections. Technical report, 2011. 10.5170/CERN-2011-002. arXiv:1101.0593

[83] Butterworth J (2016). PDF4LHC recommendations for LHC Run II. J. Phys. G.

[84] CMS Collaboration, Measurement of the inclusive W and Z production cross sections in pp collisions at $$\sqrt{s}=7\text{ TeV }$$. JHEP **10**, 132 (2011). 10.1007/JHEP10(2011)132. arXiv:1107.4789

[85] ATLAS Collaboration, Measurement of the inelastic proton-proton cross section at $$\sqrt{s} = 13\text{ TeV }$$ with the ATLAS detector at the LHC. Phys. Rev. Lett.** 117** (2016) 182002. 10.1103/PhysRevLett.117.182002. arXiv:1606.02625 10.1103/PhysRevLett.117.182002 27834993

[86] CMS Collaboration, *CMS luminosity measurements for the 2016 data taking period*. CMS Physics Analysis Summary CMS-PAS-LUM-17-001 (CERN, Geneva, 2017). https://cds.cern.ch/record/2257069

[87] CMS Collaboration, *Simplified likelihood for the re-interpretation of public CMS results*, CMS Note 2017/001 (2017). http://cdsweb.cern.ch/record/2242860

[88] Junk T (1999). Confidence level computation for combining searches with small statistics. Nucl. Instrum. Meth. A.

[89] Read AL (2002). Presentation of search results: the $$CL_{s}$$ technique. J. Phys. G.

[90] Cowan G, Cranmer K, Gross E, Vitells O (2011). Asymptotic formulae for likelihood-based tests of new physics. Eur. Phys. J. C.

[91] ATLAS and CMS Collaborations, *LHC Higgs Combination Group, Procedure for the LHC Higgs boson search combination in Summer 2011*. Technical Report ATL-PHYS-PUB-2011-11, CMS NOTE 2011/005, (2011)

[92] CRESST Collaboration, Results on light dark matter particles with a low-threshold CRESST-II detector. Eur. Phys. J. C ** 76** (2016) 25. 10.1140/epjc/s10052-016-3877-3. arXiv:1509.01515

[93] SCDMS Collaboration, New results from the search for low-mass weakly interacting massive particles with the CDMS low ionization threshold experiment. Phys. Rev. Lett. **116**, 071301 (2016). 10.1103/PhysRevLett.116.071301. arXiv:1509.02448 10.1103/PhysRevLett.116.071301 26943526

[94] PandaX-II Collaboration, Dark matter results from 54-ton-day exposure of PandaX-II experiment (2017). arXiv:1708.06917 10.1103/PhysRevLett.119.181302 29219592

[95] LUX Collaboration, Results from a search for dark matter in the complete LUX exposure. Phys. Rev. Lett.**118**(2), 021303 (2017). 10.1103/PhysRevLett.118.021303. arXiv:1608.07648 10.1103/PhysRevLett.118.021303 28128598

[96] XENON Collaboration, First dark matter search results from the XENON1T experiment (2017). arXiv:1705.06655. (**Submitted to Phys. Rev. Lett**) 10.1103/PhysRevLett.119.181301 29219593

[97] PICASSO Collaboration, Final results of the PICASSO dark matter search experiment. Astropart. Phys.** 90**, 85 (2017). 10.1016/j.astropartphys.2017.02.005. arXiv:1611.01499

[98] PICO Collaboration, Dark matter search results from the PICO-60 C$$_3$$F$$_8$$ bubble chamber. Phys. Rev. Lett. **118**, 251301 (2017). 10.1103/PhysRevLett.118.251301. arXiv:1702.07666 10.1103/PhysRevLett.118.251301 28696731

[99] S-K Collaboration, Search for neutrinos from annihilation of captured low-mass dark matter particles in the Sun by Super-Kamiokande. Phys. Rev. Lett. **114**, 141301 (2015). 10.1103/PhysRevLett.114.141301. arXiv:1503.04858 10.1103/PhysRevLett.114.141301 25910107

[100] IC Collaboration, Search for annihilating dark matter in the Sun with 3 years of IceCube data. Eur. Phys. J. C **77**, 146 (2017). 10.1140/epjc/s10052-017-4689-9. arXiv:1612.05949

[101] IC Collaboration, Improved limits on dark matter annihilation in the Sun with the 79-string IceCube detector and implications for supersymmetry. JCAP **04**, 022 (2016). 10.1088/1475-7516/2016/04/022. arXiv:1601.00653

