# Supplementary material for: Search for new physics in events with a leptonically decaying Z boson and a large transverse momentum imbalance in proton–proton collisions at √s = 13TeV
Source: Eur Phys J C Part Fields. 2018 Apr 11;78(4):291. doi: 10.1140/epjc/s10052-018-5740-1 (PMC6445555; doi:10.1140/epjc/s10052-018-5740-1)
Supplement: Supplementary file 1 — Supplementary material 1 (pdf 207 KB) [file 10052_2018_5740_MOESM1_ESM.pdf]

e-mail: cms-publication-committee-chair@cern.ch

Key words. CMS, Physics, Exotica, dark matter, simplified model, invisible Higgs

Search for new physics in events with a leptonically decaying Z boson and a large transverse momentum imbalance in proton-proton collisions at $\sqrt{s} = 13\,\mathrm{TeV}$: Supplementary material

The CMS Collaboration

CERN

April 2, 2018

Abstract. Supplementary material in support of the search for new physics in events with a Z boson produced in association with large missing transverse momentum at the LHC based on the 2016 data sample of proton-proton collisions recorded with the CMS experiment at $\sqrt{s} = 13\,\mathrm{TeV}$.

Figure 1 shows the correlations between the estimated background yields in $p_{\mathrm{T}}^{\mathrm{miss}}$ bins in the signal region. These correlations can be used in conjunction with the total background estimates in the signal region from the control-region-only fit to re-construct an approximate likelihood function for this analysis. Using this likelihood function, together with an alternative signal model, a fit can be performed to the observed data to recast the results in the simplified likelihood framework [1]. To utilize the simplified likelihod method, a prediction of the reconstructed event yields in each $p_{\mathrm{T}}^{\mathrm{miss}}$ bin is required. This is best obtained by using a detector simulation program such as DELPHES, however a reasonable prediction can be obtained by: applying a generator-level selection that parallels the reconstruction-level selection described in Section 7, omitting tau lepton and b jet vetoes; smearing the $p_{\mathrm{T}}^{\mathrm{miss}}$ with a Gaussian kernel of 24 GeV width; and scaling by a reconstruction efficiency of 0.70.

Figure 2 shows the 95% CL upper limits on the SM-like Higgs boson branching fraction to invisible particles, as a function of its mass. The Higgs production cross section assumed in this figure includes only the $q\overline{q} \to Z(\ell\ell)H$ process.

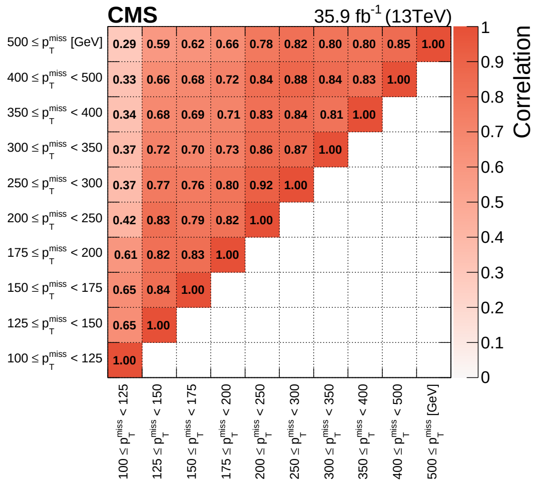

Fig. 1. Correlations between the estimated background yields in the signal region $p_{\mathrm{T}}^{\mathrm{miss}}$ bins. The correlations are obtained after performing a combined fit to data in all control regions, but excluding data in the signal region. Since the correlation matrix is symmetric by construction, the part below the diagonal is not shown.

References

1. CMS Collaboration, "Simplified likelihood for the re-interpretation of public CMS results", CMS Note 2017/001, 2017.

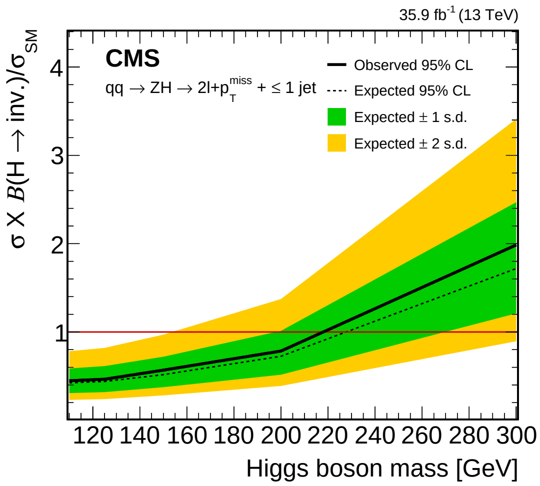

Fig. 2. Expected and observed 95% CL upper limits on $\mathcal{B}(\mathrm{H} \to \mathrm{inv.})$, assuming SM Higgs boson production cross sections, as a function of the Higgs boson mass.
